# An Integrated Analysis Identified TAGLN2 As an Oncogene Indicator Related to Prognosis and Immunity in Pan-Cancer

**DOI:** 10.7150/jca.84454

**Published:** 2023-06-19

**Authors:** Teng Pan, Shubin Wang, Zhiyu Wang

**Affiliations:** Department of Immuno-Oncology, The Fourth Hospital of Hebei Medical University, Shijiazhuang, China.

**Keywords:** TAGLN2, pan-cancer, prognosis, tumor microenvironment, immunity, programmed cell death

## Abstract

**Background:** Transgelin-2 (TAGLN2) has long been regarded as an actin-binding protein that modulates actin gelation and controls actin cytoskeleton dynamics. However, recent studies have reported that TAGLN2 can directly or indirectly participate in multiple cancer-related processes, including cell migration, proliferation, differentiation, and apoptosis. To further investigate the role of TAGLN2 in carcinogenesis, a comprehensive analysis was launched to evaluate the expression status and prognostic value of TAGLN2 in pan-cancer.

**Methods:** Herein, data was retrieved from publicly online websites and databases, including The Cancer Genome Atlas (TCGA), Genotype-Tissue Expression (GTEx), Cancer Cell Line Encyclopedia (CCLE), UCSC Xena, cBioPortal, Human Protein Atlas (HPA), TIMER2.0, CancerSEA, GDSC, and ImmuCellAI. Gene expression pattern and its correlation with prognosis were assessed across cancer types. Moreover, an analysis was conducted to explore the relationships between TAGLN2 and methylation, copy number values (CNVs), tumor microenvironment (TME), immune cell infiltration, immune-relevance genes, tumor mutation burden (TMB), microsatellite instability (MSI), and IC50. Additionally, R package “clusterProfiler” was utilized to perform enrichment analysis on TAGLN2. Finally, the ability of TAGLN2 as an oncogene was preliminarily verified in vitro in UCEC.

**Results:** Our findings revealed that TAGLN2 was specifically overexpressed and related to an unfavorable prognosis in most cancers. There was a significant connection between TAGLN2 expression and methylation and CNVs. Besides, we identified TAGLN2 correlated to TME, immune cell infiltration, immune-relevant genes, TMB, and MSI, suggesting an immunoregulatory role in cancers. Notably, TAGLN2 expression showed a positive correlation with macrophages, and cancer-associated fibroblasts, whereas a negative correlation with the infiltration degree of B cells. Mechanically, the results obtained from Gene Set Enrichment Analysis (GSEA) and Gene Set Variation Analysis (GSVA) provided theory-supportive evidence that TAGLN2 interlinkages with immunity and programmed cell death. Overall, anti-tumor drugs were overtly associated with TAGLN2 dysregulation among diverse cancers. At last, UCEC cell lines with TAGLN2-depleting had an inhibition of the migration and invasion ability.

**Conclusions:** These findings enriched the knowledge about the role of TAGLN2 in tumorigenesis and progression, revealing TAGLN2 may serve as a potential therapeutic strategy for various malignancies.

## Introduction

The global cancer burden continues to increase with substantial mortality^1^. Cancer treatment has been always considered one of the most critical and vital themes of clinical issues. Many approaches have been developed, depending on the type and the stage of the tumor^2^. Targeted and immune-based therapies have already transformed the standard-of-care for several malignancies^3^. However, there are still important challenges in the field of cancer therapy, on the one hand, druggable genomic alterations are diverse, and tumor heterogeneity and acquired resistance are probably the main limitations for effective targeted therapy. Besides, the long-term survival benefits of immune checkpoint inhibitors are restricted to a minority of patients^4^. Therefore, predictive markers with robustly validated are needed to help us optimize treatment delivery and selection.

Actin-binding proteins (transgelins, TAGLNs) are regulated by alternative splicing, producing multiple transcripts, consisting of TAGLN1, TAGLN2, and TAGLN3^5^. Transgelins are a kind of protein that regulates actin polymerization, aggregating (or bundling), or cross-linking^6^. Of note, as a protein affecting dynamics of the actin cytoskeleton via stabilization of actin filaments, TAGLN2 is also both directly and indirectly involved in many cancer-related processes such as migration, proliferation, differentiation, or apoptosis^7^. A previous report revealed that TAGLN2 could promote the proliferation, invasion, migration, and epithelial-mesenchymal transition(EMT) of colorectal cancer cells by activating STAT3 and regulating ANXA2 expression^8^. In Wang's study, TAGLN2 may promote the invasion of papillary thyroid cancer cells via the Rap1/PI3K/AKT signaling pathway. More importantly, the existing study provided important evidence that hypoxia-inducible TAGLN2 was involved in the selection of cancer cells with enhanced EMT properties to overcome the detrimental environment of cancer cells^9^. What is more, TAGLN2 was interlinked with the function of dendritic cells^10^, cytotoxic T cells^11^, and macrophages^12^. In recent years, the TAGLN2 gene has attracted much interest and attention, leading to further exploration and investigation into its possible applications. However, thorough research on TAGLN2 is lacking in pan-cancer.

The emergence of high-throughput omics data and the development of bioinformatics have revealed a large number of potential biomarkers and patterns. Bioinformatics technology has been rapidly developed and applied, which has greatly improved the level of physiological mechanism research, the accuracy of disease diagnosis and treatment, and the targeting of drug application, making biomedical research and application enter the era of digitalization and simulation.

**Figure 1:**
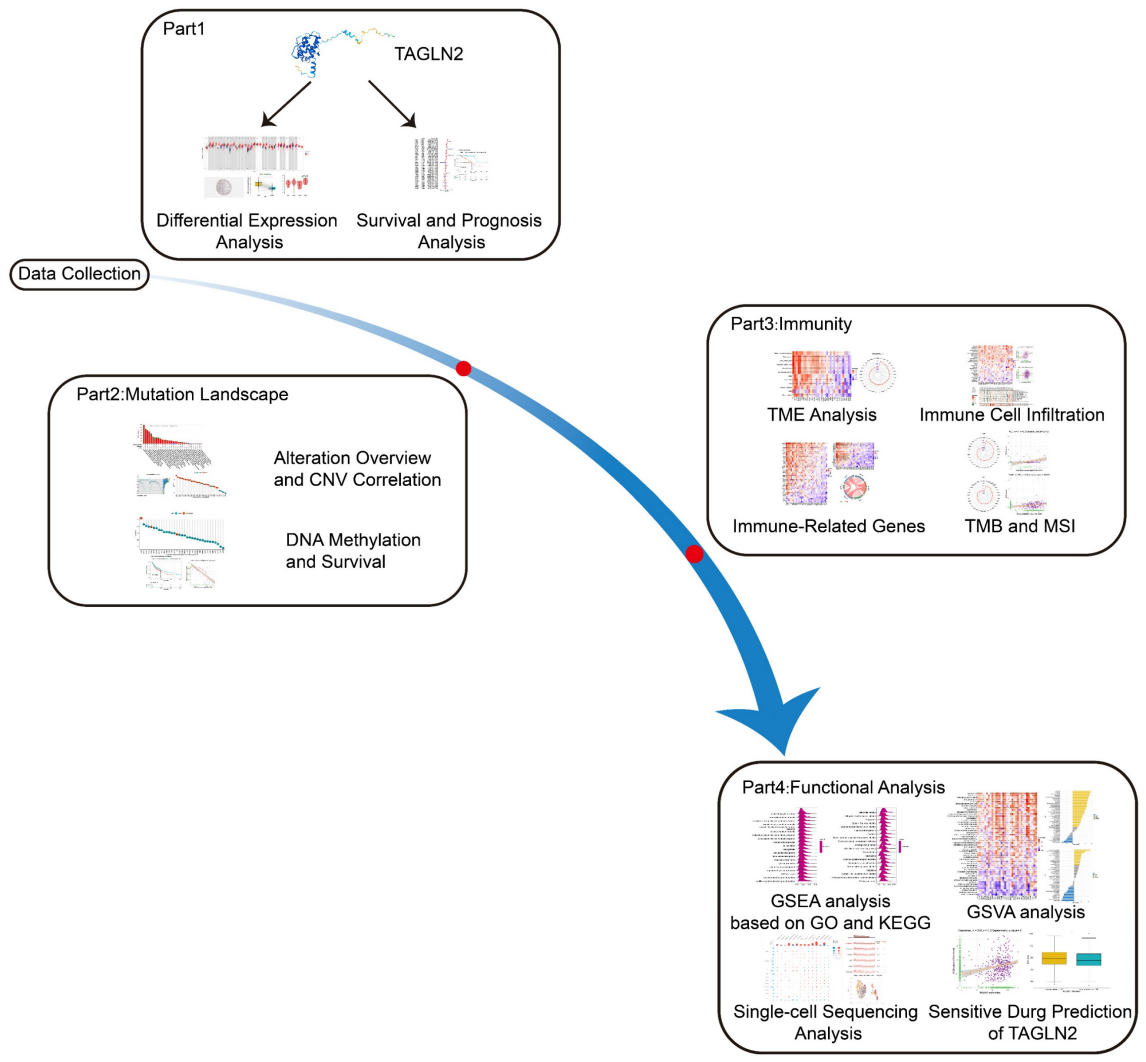
A study workflow of systematic pan-cancer analysis of TAGLN2.

In our study, utilizing bioinformatics methods, we carried out an in-depth pan-cancer analysis on TAGLN2, comprising of expression level, prognostic traits, alteration, DNA methylation, and functional enrichment analysis. At the same time, the correlations between TAGLN2 expression and immune-related genes, immune cell infiltration, and drug sensitivity were further evaluated. Overall, the results indicated a higher expression of TAGLN2 in most malignancies, which indicated a poor prognosis. Its possible oncogenic mechanisms in cancer development might be relevant to the tumor immune microenvironment, programmed cell death, and drug resistance. The detailed flow diagram of the research can be found in **Figure 1**. To sum up, our pan-cancer analysis of TAGLN2 identified that it might function as a unique indicator for clinical prognosis prediction and is inextricably connected with the tumor immune microenvironment.

## Materials and Methods

### Data Collection and Processing

The TCGA, GTEx, and CCLE transcriptome profiling and associated clinical data were obtained from UCSC XENA website (https://xenabrowser.net/datapages). We evaluated the expression levels of TAGLN2 in 31 normal tissues, 33 tumor tissues, and multiple cancer cell lines by exploiting the downloaded data. Further, to effectively address the shortcoming of limited normal tissue data in many TCGA cohorts, we combined the normal tissue data from GTEx database and TCGA tumor tissue data to explore the differential expression of 33 tumors. We incorporated all available tumor tissue samples from the TCGA database, and the detailed information for each of the tumor tissues can be found on the website (https://xenabrowser.net/datapages/?dataset=Survival_SupplementalTable_S1_20171025_xena_sp&host=https%3A%2F%2Fpancanatlas.xenahubs.net&removeHub=https%3A%2F%2Fxena.treehouse.gi.ucsc.edu%3A443). Subsequently, we selected samples with transcriptome data available, resulting in a final sample size presented in **Table S2**. TAGLN2 expression data were normalized by log2 conversion. Next, the expression of TAGLN2 in different cancer types across different pathological stages according to the World Health Organization (WHO), as well as its paired differential expression, has been visualized in the form of violin plots and box plots. Subsequently, immunohistochemistry (IHC)-based protein expression images of TAGLN2 protein expression in clinical specimens of cancer patients were collected from the HPA database (https://www.proteinatlas.org/). In addition, the HPA database had prepared us with the gene-location cell pattern map to acknowledge the TAGLN2 distribution.

### Genetic Alteration Landscape

The cBioPortal (http://www.cbioportal.org/) was a comprehensive cancer genomics resource database that provided a wealth of useful information, such as the “Cancer Types Summary”, which provided an overview of the types and frequencies of mutations in the cancer genome; the “mutations” panel displayed mutation sites in the Pfam protein domain of the target gene; “View 3D Structure” allowed us to view the location of mutation sites in the three dimension (3D) structure, and the “Plots” module displayed the findings of mRNA expression and RSEM (batch normalized from Illumina HiSeq RNASeqV2) throughout all TCGA tumors. Download gene mutation data of each tumor from the UCSC XENA database and use the R package "maftools" to construct waterfall plots to display the somatic mutation landscape based on TAGLN2 expression, as well as forest plots to show the difference of gene mutation.

Besides, CNVs and promoter methylation data were also retrieved from the cbioportal database, the Pearson correlations between TAGLN2 expression and CNVs/promoter methylation in each kind of tumor were calculated, at last, the correlations in pan-cancer were visualized in the lollipops. Furthermore, the effect of methylation on prognosis was also checked by applying the “survival” R packages to plot the Kaplan-Meier curves, which set medium cutoff as the cutoff value.

### Correlation Analysis of Prognosis

To evaluate the relationship between TAGLN2 expression and patients' prognosis, the pan-cancer samples were separated into TAGLN2 high- and low- expression groups with the medium cutoff as the cutoff value. The R-packages “survival” and “forestplot” were employed to conduct univariate Cox analysis, namely overall survival (OS), disease-free interval (DFI), disease-specific survival (DSS), and progression-free interval (PFI). Subsequently, the R-packages “survminer” and “survival” were adopted to generate Kaplan-Meier curves.

### Relevance Between TAGLN2 Expression and Tumor Microenvironment

The constant interactions between tumor cells and the tumor microenvironment play decisive roles in tumor initiation, progression, metastasis, and response to therapies^13^. Therefore, TAGLN2 expression and TME-related gene signature scores were examined using R packages according to a previously reported method^14^. Based on R packages “ESTIMATE”, Stromalcore and ImmuneScore specified the presence of stromal cells and the level of infiltrating immune cells in each tumor sample, forming the basis of ESTIMATEScore to evaluate the tumor purity^15^.

### Immune Cell Infiltration Analysis

The tumor immune microenvironment (TIME) mainly consists of distinct immune cell populations in tumor islets and is highly associated with the antitumor immunological state in the TME^16^. We performed Pearson correlation analysis on immune cell infiltration fetched from a total of 3 data sources, one was Table S1 in the published article^17^, and another from the ImmuCellAI database (http://bioinfo.life.hust.edu.cn/ImmuCellAI#!/), both of which were analyzed with the CIBERSORT tool. Eventually, TIMER2.0(http://timer.cistrome.org/) provided a more robust estimation of immune infiltration levels for TCGA tumor profiles using six state-of-the-art algorithms, namely, CIBERSORT, CIBERSORT_ABS, EPIC, MCPCOUNTER, QUANTISEQ, TIMER as well as XCELL^18^. Together, a comprehensive analysis and visualization of tumor-infiltrating immune cells were provided.

### Association Between TAGLN2 and Immune-Related Genes

For the aim of identifying the relevance between TAGLN2 expression and immune-associated genes, such as immune-activating genes, immunosuppressive genes, chemokine genes, chemokine-receptor genes as well as major histocompatibility complex (MHC) genes, R packages “limma”, “reshape2”, and “RColorBreyer” were applied to conduct the investigation. Simultaneously, co-association TAGLN2 with the major immune checkpoint genes was performed and ultimately presented in the form of chord diagrams.

### Correlation of TAGLN2 With TMB and MSI

The somatic mutation data of tumor mutation burden were acquired from the Genomic Data Commons (GDC) data portal website (https://portal.gdc.cancer.gov/) of the UCSC Xena repository and then managed with R package “mafTools” for statistical analysis. And the microsatellite instability data were obtained according to a previously published report^19^. Finally, the results were visualized in the form of radar plots, and scatter plots.

### Functional Enrichment Analysis

Enrichment analysis helps researchers discover novel biological functions, genotype-phenotype relationships and disease mechanisms^20^. To gain a comprehensive understanding of the underlying mechanisms of TAGLN2, GSEA was launched to investigate the potential signal paths of TAGLN2 in the tumor process by using “clusterProfiler”. The 20 highest-ranked terms from Gene Ontology(GO) and Kyoto Encyclopedia of Genes and Genomes(KEGG) pathways with adjusted P<0.05 were displayed. With regard to GSVA, the MSigDB database (https://www.gsea-msigdb.org/gsea/msigdb/index.jsp) was consulted to calculate the 50 best-characterized pathway scores of each sample.

Regulated cell death (RCD) or programmed cell death (PCD) is not only essential in embryonic development but also plays an important role in the occurrence and progression of diseases, especially cancers. Escaping of cell death is one of the hallmarks of cancer^21^. Hence, we performed correlation analysis of TAGLN2 with key genes in multiple cell death modalities to explore the possible connections between TAGLN2 and programmed cell death programs, such as autophagy, ferroptosis, and pyroptosis.

### Functional States Analysis

The relevance of TAGLN2 across 14 functional states in distinct cancers was plotted at a single-cell level through the “correlation plot” module of the CancerSEA website (http://biocc.hrbmu.edu.cn/CancerSEA/home.jsp). And correlations between TAGLN2 and functional states in indicated single-cell datasets were depicted. T-SNE diagrams were obtained to describe the distribution of cells, every point represented a single cell, and the color of the point represented the expression level of TAGLN2 in the cell.

### Drug Sensitivity Analysis

On the basis of Spearman correlation analysis, the relationship between TAGLN2 expression and the half-maximal inhibitory concentrations (IC50) of 192 compounds was evaluated through the GDSC (https://www.cancerrxgene.org/) portal. Further, the expected medication response differences for each drug in the TAGLN2 high- and low-expression groups were elucidated.

### Clinical specimens

Six cases of UCEC tissues and paired adjacent non-tumor tissues were collected from the Fourth Affiliated Hospital of Hebei Medical University. All the patients received a UCEC diagnosis with the results of the histopathological examination. The hospital ethics committee approved the ethical consent.

### Cell Culture

UCEC cell lines hEEC, ISHIKAWA, and KLE were purchased from the China Center for Type Culture Collection (CCTCC) and were cultivated in DMEM (11995, Solarbio) with 10% FBS (Invitrogen) at 37℃ incubator filled with 5% CO_2_.

### Immunofluorescence Assay

Cell immunofluorescence staining is a technique used to identify specific proteins in cells. The process began by fixing the cells on a slide and permeabilizing the cells with a detergent (T8200, Solarbio). An antibody specific to the desired protein (1:50, sc-373928, SANTA CRUZ) was then added and bound to the protein. After washing away unbound antibodies, a fluorescently labeled secondary antibody (1:600, Cat#: A23310, Abbkine) was added and bound to the primary antibody. Next, cells were stained by DAPI (S2100, Solarbio) for 30s, away from dark. The slide is then washed and viewed under a fluorescent microscope (Nikon, Tokyo, Japan).

### RNA extraction and qRT-PCR assay

According to TRIzol reagent instructions (SolarBio), total RNA was extracted from cells and tissues. Following the guidelines of Transcriptor First Strand cDNA Synthesis Kit (Takara), RNA was reverse‐transcribed into complementary DNA (cDNA). The real‐time reverse‐transcription polymerase chain reaction(qRT‐PCR) was carried out by the use of the GoTaq® qPCR Master Mix (Promega). The GAPDH was used as endogenous control, and an optimized comparative Ct (2-^△△Ct^) value method was used to measure the relative expression level. The samples were tested in triplicate. The sequences of primers were as follows: TAGLN2: F: AGTGACATTCCCAGAGAGCC; R: GGCCCCTAAATTTTGGTCCC. GAPDH: F: TGTGGGCATCAATGGATTTGG; R: ACACCATGTATTCCGGGTCAAT.

### Cell transfections

ISHIKAWA cells were grown to 80%-90% confluence in 6-well plates and then transfected with Si-TAGLN2-1 and Si-TAGLN2-2 to knock down TAGLN2 expression according to the manufacturer's protocol using Lipofectamine 2000 (Invitrogen). The SiRNAs of TAGLN2 were synthesized by Genepharma and the sequences for TAGLN2 SiRNAs were as follows: Si-TAGLN2-1: GCAAGAACGUGAUCGGGUUTT; Si-TAGLN2-2: CUGAGCGCUAUGGCAUUAATT; Si-NC: UUCUCCGAACGUGUCACGUTT. Fluorescence microscopy was used to calculate the fluorescein‐labeled cells. The western blot was performed to verify the transfection downregulating the level of TAGLN2 protein.

### Wound-healing assay

A wound was made by scratching the cell culture surface with a 200 μl pipette 24 hours after transfection. Phase contrast images of the wound were measured at 0h and 24h after the scratch. Three separate experiments were performed, with cells transfected with Si-NC serving as the control.

### Transwell assay

The transfected cells were inoculated in 1×105 cells into the upper chamber, supplemented with 200 μl serum‐free medium and 600 μl medium containing 10% FBS in the lower compartment. After 24 h of incubation at 37°C, the cells in the upper chamber were wiped off, while the invasive cells located in the lower chamber were fixed with 4% paraformaldehyde and stained with 1% crystal violet for 20 min. Of note, depending on the experimental design, matrigel could be present or absent. Count invading cells in five randomly selected microscope fields.

### Western blotting analysis

The total protein was collected in RIPA buffer (Beyotime, Shanghai, China), and after that centrifuged at 4°C for 10 min. Then we would collect the supernatant and calculate the protein concentration using a BCA kit (Beyotime, Shanghai, China). The protein samples were electrophoresed on sodium dodecyl sulfate-polyacrylamide (SDS-PAGE) gel and then transferred to a polyvinylidene fluoride (PVDF) membrane. At room temperature, 5% milk powder was sealed for 2 h and incubated with primary antibody overnight at 4°C. The antibodies used were as follows: GAPDH (1:10000, 10494-1-AP, Proteintech); TAGLN2 (1:500, sc-373928, SANTA CRUZ). On the next day, after washing three times with TBST, they were incubated with secondary antibodies for 1 h at room temperature. The signal was visualized using an enhanced ECL reagent (Multi Sciences).

### Statistical Analysis

All gene expression data were normalized through a log2 transformation for the subsequent analyses. We calculated the correlation between two variables utilizing Spearman's or Pearson's method. The differences between two groups were analyzed via Student's t-test or Wilcoxon rank sum test. One-factor Analysis of Variance(One-way ANOVA) analyses of variance was used to calculate statistical significance more than two groups. The Kaplan-Meier curves and univariate Cox proportional hazard regression models were applied to all survival analyses. R (Version 4.1.2) and Rstudio software were exploited to perform statistical analysis of the bioinformatics results. And graphs were plotted using GraphPad Prism9.0. Quantitative data analysis was performed with the open-source software “ImageJ”. A two-tailed P value of <0.05 indicated statistically significant. *, P < 0.05; **, P < 0.01; and ***, P < 0.001, respectively.

## Results

### Abnormal Expression Levels of TAGLN2 in Various Malignancies

**Figure 2:**
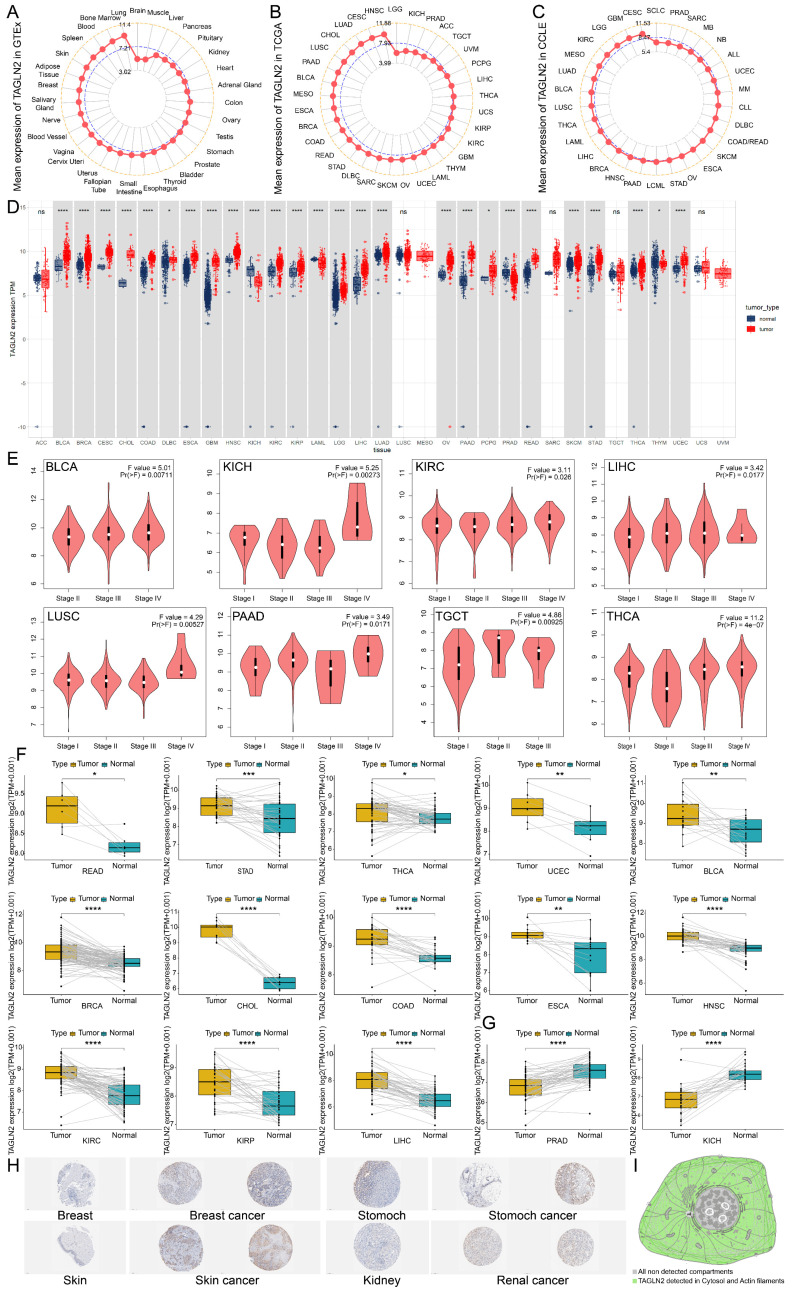
TAGLN2 expression profiles in normal tissues and cancers. (A, B) TAGLN2 expression levels in tumor tissues from TCGA database(A) and in normal tissues from GTEx database(B). (C) TAGLN2 expression levels in tumor cell lines from CCLE database. (D)TAGLN2 expression difference between tumor tissues from TCGA database and normal tissues from the GTEx database. (E) The expression levels of TAGLN2 based on the different pathological stages analyzing using GEPIA2.0. (F-G) Paired differential analysis of TAGLN2 expression in matched tumor and adjacent normal tissues from TCGA database. (H) The immunohistochemistry images of TAGLN2 in different normal(left) and paired tumor(right) tissues from HPA database. (I) Schematic diagram of distribution of TAGLN2 from HPA database. * p-value < 0.05, ** p-value < 0.01, *** p-value < 0.001, and **** p-value < 0.0001.

To begin with, we attempted to analyze the expression pattern of TAGLN2 in various normal/tumor tissues and cancer cells. The physiological gene profiles of TAGLN2 generated by the GTEx project reported the highest expression in lung tissues among various normal tissues, but most other normal samples had low levels of TAGLN2 expression **(Figure 2A)**. However, according to the TCGA dataset, TAGLN2 mRNA expression was highly expressed in a variety of malignancies, including HNSC, CESC, LUAD, and CHOL, but low in LGG **(Figure 2B)**. Moreover, in the CCLE database, mRNA expression data across 30 cancer cell lines were examined. Cancer cells had approximately two-fold increase in TAGLN2 expression compared with corresponding normal tissues **(Figure 2C)**. Besides, given the limited number of normal samples in TCGA dataset, and to enhance the comparison, we merged the normal tissue data from GTEx database and TCGA tumor tissue data to explore the expression of 33 tumors **(Figure 2D)**. The results revealed that the TAGLN2 mRNA expression was significantly increased in 23 distinct types of tumors, including BLCA, BRCA, CESC, CHOL, COAD, DLBC, ESCA, GBM, HNSC, KIRC, KIRP, LGG, LIHC, LUAD, OV, PAAD, PCPG, READ, SKCM, STAD, THCA, and UCEC. Oppositely, TAGLN2 expression level was downregulated in KICH, LAML, PRAD, and THYM. Nevertheless, the expression of TAGLN2 in ACC, LUSC, MESO, SARC, TGCT, UCS, and UVM did not differ significantly.

Following that, we examined the association between TAGLN2 expression and the pathological stages of different cancers and found that TAGLN2 expression varied across different stages of BLCA, KICH, KIRC, LIHC, LUSC, PAAD, TGCT, and THCA** (Figure 2E)**, though no significant correlation was observed in other tumor types** (Figure S1A)**. We proceeded to perform paired differential expression analysis of TAGLN2 between tumor and normal tissues within TCGA and discovered that TAGLN2 was upregulated in BLCA, BRCA, CHOL, COAD, ESCA, HNSC, KIRC, KIRP, LIHC, READ, STAD, UCEC, and THCA when compared to their matched normal samples **(Figure 2F)**, and decreased expression in PRAD and KICH was further confirmed** (Figure 2G)**. Next, we evaluated TAGLN2 protein expressions based on the HPA database, which displayed the IHC staining results of TAGLN2 protein in tumor and normal tissues. The analysis revealed that TAGLN2 expression in normal breast, stomach, skin, kidney **(Figure 2H)**, cervix, colon, endometrium, nasopharynx, liver, lung, ovary, pancreas, and urinary bladder** (Figure S1B)** tissues were weak or negative, whereas in the corresponding tumor tissues, such expressions were moderate or strong. Furthermore, a diagram of the cellular distribution of TAGLN2 indicated that TAGLN2 protein was mainly localized to the cytosol, with some additional localization to the actin filaments **(Figure 2I)**. Thus, the results suggested that TAGLN2 expression was elevated at both mRNA and protein levels in most of the cancers surveyed.

### The Genetic Alteration Landscape and Methylation Analysis of TAGLN2 in Multiple Cancers

**Figure 3:**
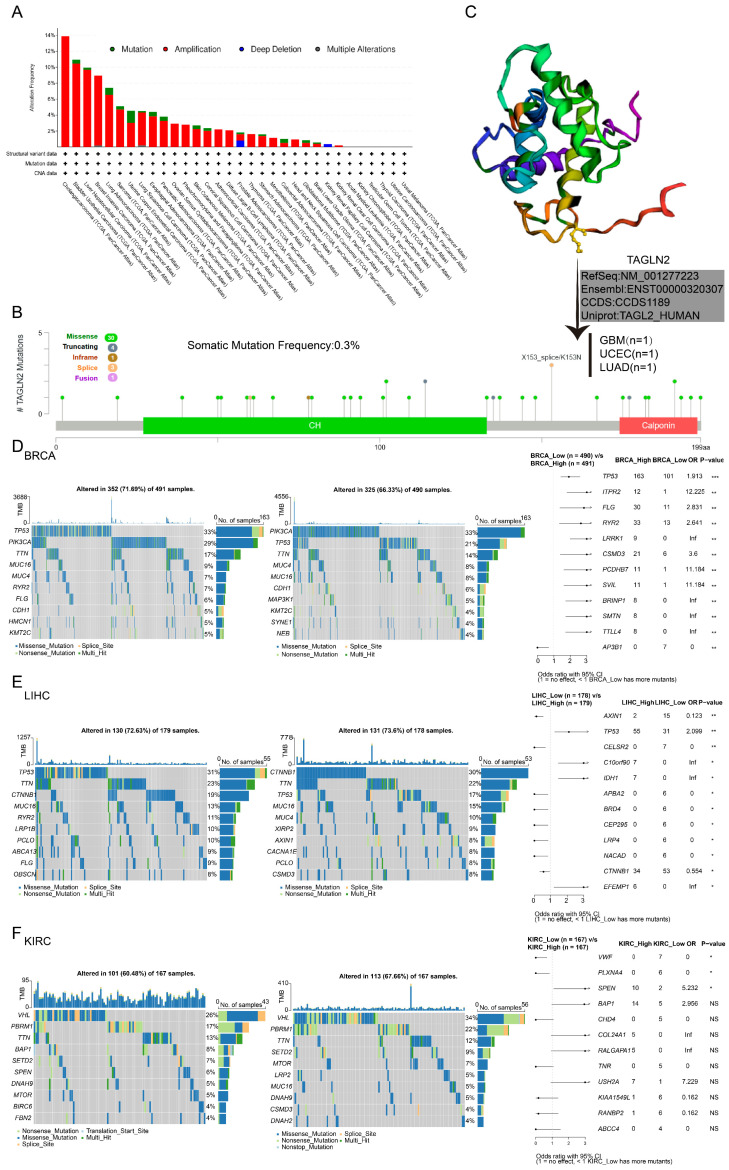
Mutation feature of TAGLN2 in different tumors revealing by use of the cBioPortal tool. (A) The alteration frequency with mutation type. (B) The alteration frequency of mutation site. (C) The mutation site with the highest alteration frequency X153_splice/K153N in the 3D structure of TAGLN2. (D-F) Waterfall plots of tumor somatic mutation in the high- and low-risk groups in BRCA(D), LIHC(E), and KIRC(F). * p-value < 0.05, ** p-value < 0.01, *** p-value < 0.001, and **** p-value < 0.0001.

Through the publically online database cBioPortal, we curated a pan-cancer analysis of TAGLN2 genetic alteration in various tumor samples from TCGA datasets** (Figure 3A)**. We discovered that genetic changes of TAGLN2 were mostly of the “amplification” type, which were significantly observed in almost all TCGA cancer cases. Mutations were likewise distributed in multiple cancers. Cholangiocarcinoma had the highest alteration frequency (13.89%), followed by bladder urothelial carcinoma (10.95%), liver hepatocellular carcinoma (9.95%), and breast invasive carcinoma (8.95%), with “amplification” as the prevalent alteration type.

It is worth noting that “mutation” was the predominant alteration type in the UCEC samples (1.51%). Moreover, “deep deletion” of TAGLN2 was observed in prostate adenocarcinoma cases (0.81%) and all kidney renal papillary cell carcinoma cases (0.35%). Furthermore, “multiple alterations” were detected in breast invasive carcinoma and lung squamous cell carcinoma, with alteration frequencies of 0.18% and 0.21%, respectively. The types, sites, and case numbers of the TAGLN2 gene modification were further depicted in **Figure 3B**. The total somatic mutation frequency was 0.3%, with missense mutations constituting the majority. Among, the X153_splice/K153N alteration was detected in 1 GBM case, 1 UCEC case, and 1 LUAD case, which could be clearly observed in the 3D structure of the TAGLN2 protein **(Figure 3C)**. And as stated in **Figure S2A,** the types of TAGLN2 gene alterations were diverse, resulting in changes in gene expression. As illustrated in **Figure 3D-F and Figure S2B**, the patients were categorized into two groups based on the median of TAGLN2 expression: the high-expression group and the low-expression group. We then analyzed the differently mutated genes between the two groups, and the results showed the top mutations occurred in BRCA, LIHC, KIRC, ESCA, PAAD, and UCEC. Intuitively, TTN, TP53, and MUC16 were the most commonly mutated genes.

**Figure 4:**
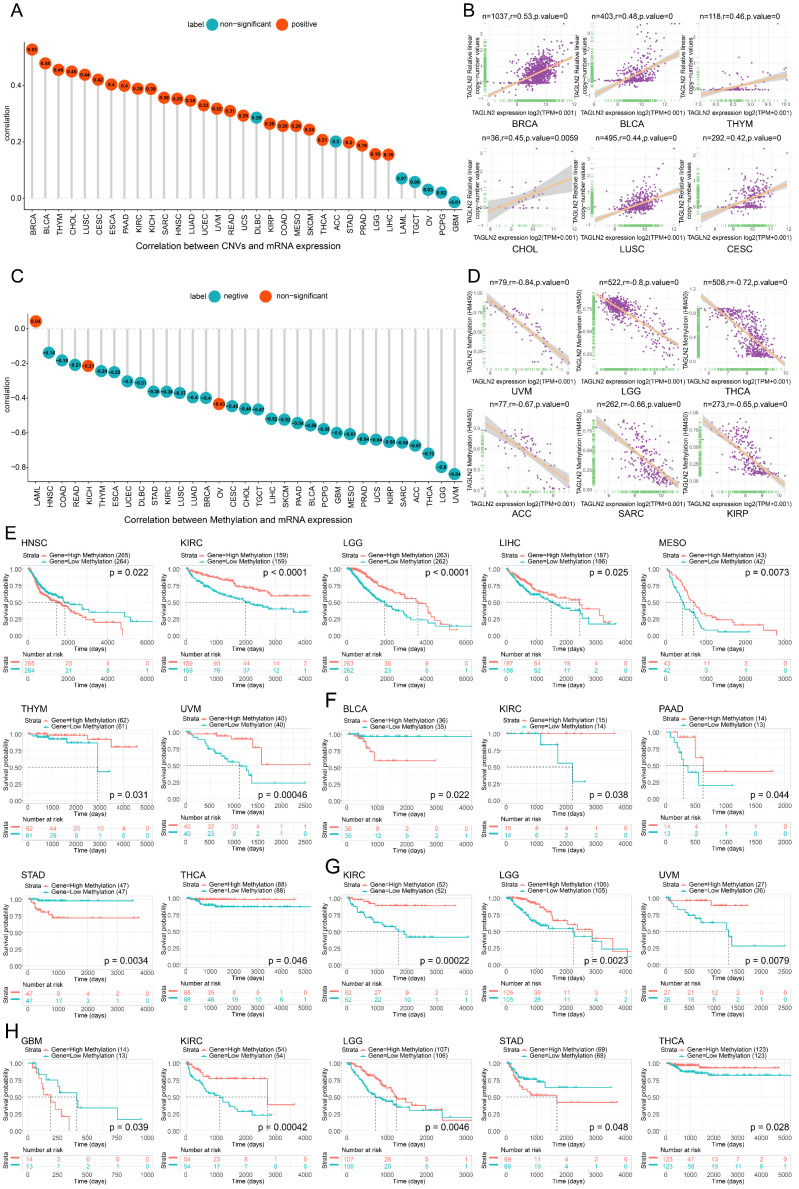
The correlation between TAGLN2 expression and CNVs and methylation. (A) The correlation between TAGLN2 expression and CNVs. (B) The top 6 with the highest pearson correlation scores between TAGLN2 and CNVs. (C) The correlation between TAGLN2 expression and methylation. (D) The top 6 with the highest pearson correlation scores between TAGLN2 and methylation. (E-H) Kaplan-Meier curves illustrating the relationships between TAGLN2 methylation levels with OS(E), DFI(F), DSS(G), and PFI(H) in the indicated cancers, respectively.

We then demonstrated the Pearson correlation between TAGLN2 CNVs and mRNA expression. Copy number variations (CNVs) are a form of genetic variation, characterized by the variable number of DNA fragments in the human genome. CNVs generally range from a kilo base pairs to a mega base pairs in length, and have been found to have a significant impact on cancer biology and drug treatment^22^. As shown in **Figure 4A** where the numbers in the circles represented correlation scores, in BRCA, BLCA, THYM, CHOL, LUSC, CESC, ESCA, PAAD, KIRC, KICH, SARC, HNSC, LUAD, UCEC, UVM, READ, UCS, KIRP, COAD, MESO, SKCM, THCA, STAD, PRAD, LGG, and LIHC, there is a substantial positive connection between TAGLN2 CNVs and mRNA expression. On the contrary, this connection was not significant in ACC, DLBC, TGCT, LAML, OV, PCPG, and GBM. **Figure 4B** showed the top six with the highest correlation scores.

Similarly, the TAGLN2 DNA methylation landscape in pan-cancer was sketched. DNA methylation, as an epigenetic mechanism, occurs by adding a methyl group of cytosines in position 5 by DNA methyltransferases and abnormal methylation is well-known hallmark of cancer development and progression^23^. As illustrated by the lollipop chart in **Figure 4C**, it reflected a significant correlation between TAGLN2 expression and methylation in a total of 30 tumors. Among the vast majority of the tumors studied, the expression of TAGLN2 was Pearson negatively linked with gene promoter methylation, with the exception of LAML, KICH, and OV. **Figure 4D** showed the top six with the highest correlation scores.

In order to further explore the relationship between promoter methylation and prognosis of survival, we conducted Kaplan-Meier analysis (OS, DSS, PFI, DFI) in pan-cancer. It was observed that in patients diagnosed with HNSC, promoter hypermethylation was associated with a poorer OS **(Figure 4E)**. On the other hand, for KIRC, LGG, LIHC, MESO, THYM, and UVM, promoter hypermethylation was linked to better survival. Additionally, the DFI analysis showed that TAGLN2 methylation acted as a protective marker in KIRC, PAAD, and THCA patients, whereas a detrimental factor for BLCA and STAD **(Figure 4F)**. Besides, for PFI analysis, enhanced TAGLN2 methylation was a protective factor for KIRC, LGG, and THCA, although it was a harmful factor for GBM and STAD **(Figure 4H)**. What's more, in terms of DSS, a higher TAGLN2 methylation level distinctly tended to a better prognosis in patients experiencing KIRC, LGG, and UVM** (Figure 4G)**.

### Prognostic Significance of TAGLN2

**Figure 5:**
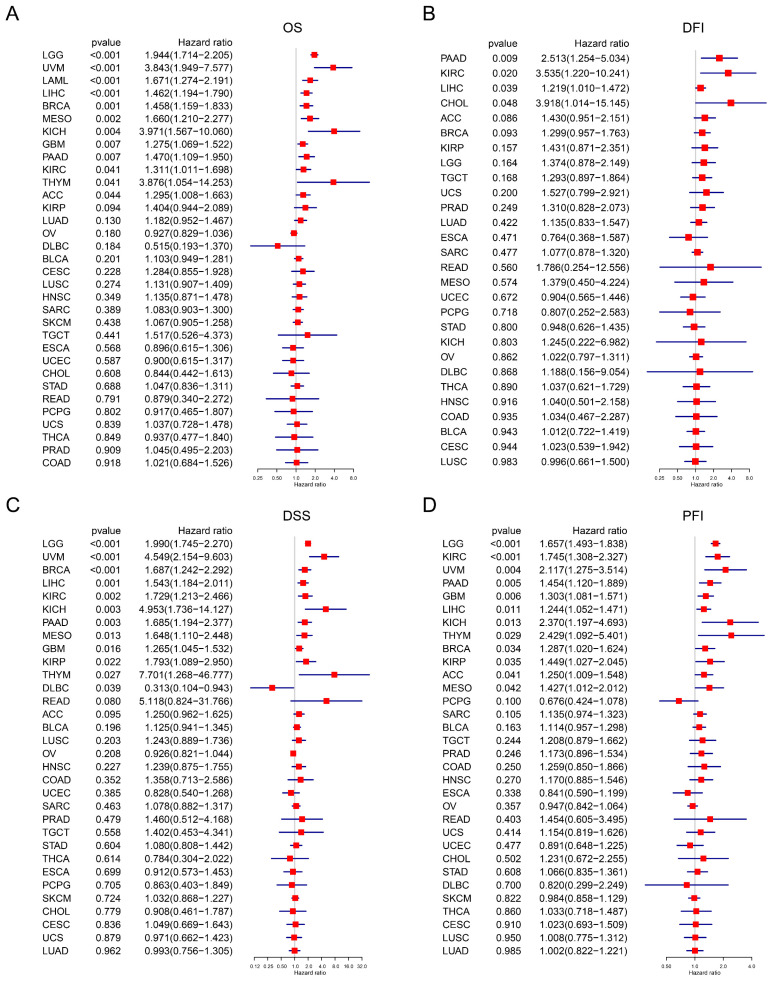
The correlation between TAGLN2 expression and prognosis by Cox regression. (A-D) Forest plots revealing the relationship between TAGLN2 and OS(A), DFI(B), DSS(C), and PFI(D) in indicated tumors, solely.

By analyzing the available data, we investigated the prognostic significance of TAGLN2 in pan-cancer. We assessed the OS, DSS, DFI, and PFI, solely. OS is defined as the time from initial diagnosis to date of death (due to any cause). Our Cox regression analysis of OS revealed that high expression of TAGLN2 was associated with shorter survival times in LGG, UVM, LAML, LIHC, BRCA, MESO, KICH, GBM, PAAD, KIRC, THYM, and ACC **(Figure 5A)**. Interestingly, similar results were obtained when we conducted the Kaplan-Meier analysis, suggesting that TAGLN2 could be a risk factor in LGG, UVM, LAML, LIHC, MESO, KICH, and ACC** (Figure S3A)**.

DFI is defined as the period between the date of diagnosis to the date of the first new tumor progression event after the patient's disease-free status (after initial diagnosis and treatment). Our analysis of DFI revealed that TAGLN2 was a high-risk gene in PAAD, KIRC, LIHC, and CHOL **(Figure 5B)**. Additionally, Kaplan-Meier plotter results showed that among individuals with PAAD, KIRC, and CHOL **(Figure S3B)**, those with high TAGLN2 expression were observed to have shorter survival times.

DSS is defined as the length of time between initial diagnosis and date of death due to the diagnosed type of cancer. For DSS, Cox regression indicated that high TAGLN2 was a risk factor for LGG, UVM, BRCA, LIHC, KIRC, KICH, PAAD, MESO, GBM, KIRP, and THYM, while individuals with DLBC had a longer survival time **(Figure 5C)**. In the Kaplan-Meier analysis, patients with increased TAGLN2 levels had poorer DSS than those with decreased TAGLN2 levels in LGG, READ, KIRC, KICH, UVM, and PAAD **(Figure S3C)**, whereas higher TAGLN2 expression was associated with better DSS in BRCA.

Finally, PFI is defined as the period from the date of diagnosis until the date of the first occurrence of a novel tumor event, which includes progression of the disease, local recurrence, distant metastasis, new primary tumor, or death due to tumor. Our PFI analysis using Cox regression identified that higher TAGLN2 expression was a risk factor for LGG, KIRC, UVM, PAAD, GBM, LIHC, KICH, THYM, BRCA, KIRP, ACC, and MESO **(Figure 3D)**. Moreover, highly expressed TAGLN2 was associated with reduced PFI in these cancers **(Figure S3D)**. Based on the above results, TAGLN2 expression is differentially correlated to the survival prognosis of patients bearing diverse cancers.

### TAGLN2 is Correlated with TME in Pan-cancer

**Figure 6:**
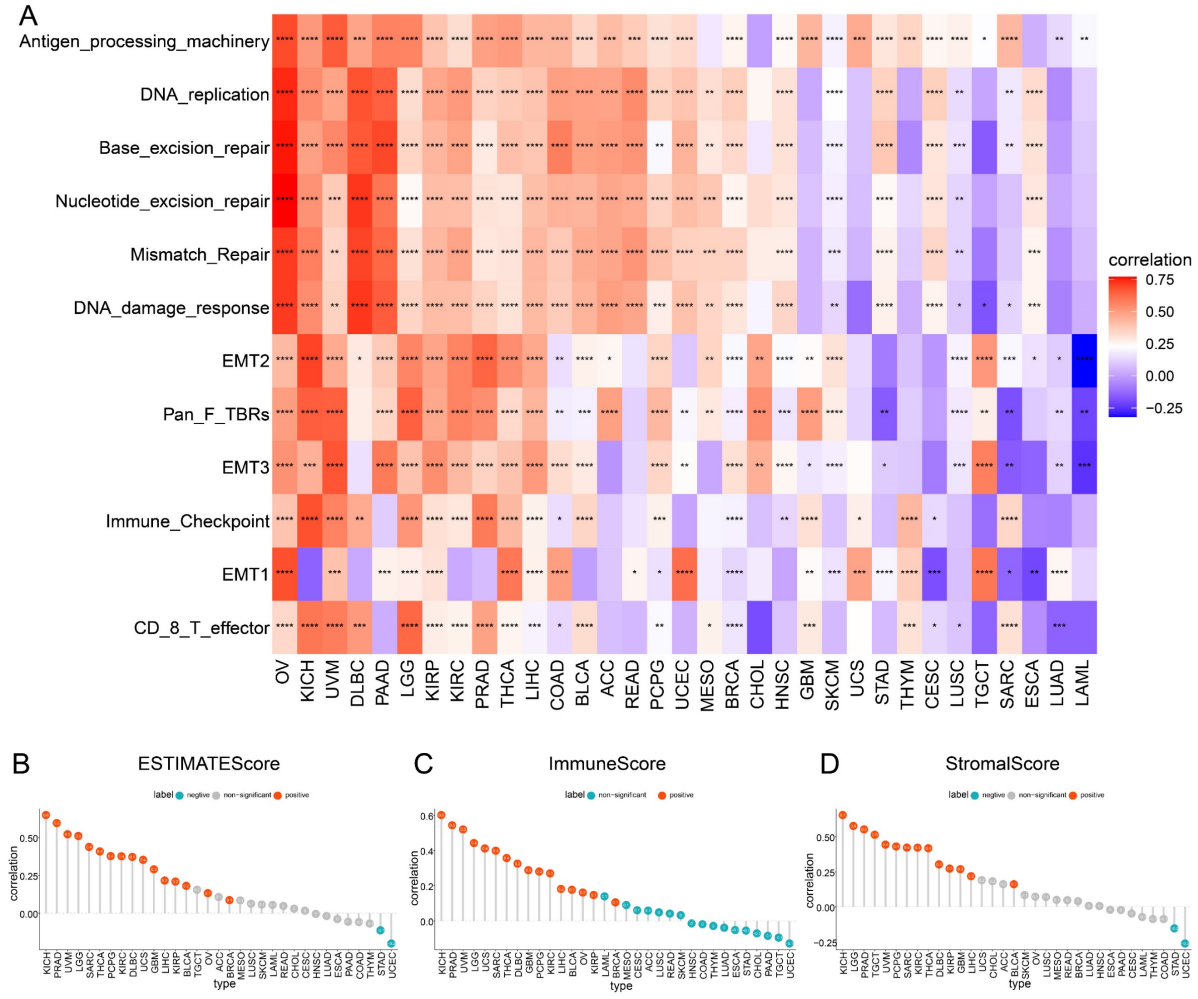
The role of TAGLN2 in tumor microenvironment. (A) Heatmap depicting the correlation strength between TAGLN2 expression and TME-related terms in pan-cancer. (B-D) The correlation of TAGLN2 expression with ESTIMATEScore(B), ImmuneScore(C), and StromalScore(D) in various cancers. * p-value < 0.05, ** p-value < 0.01, *** p-value < 0.001, and **** p-value < 0.0001.

Accumulating evidence shows that cellular and acellular components in tumor microenvironment can reprogram tumor initiation, growth, invasion, metastasis, and response to therapies^24^. Therefore, it was essential to explore the roles that TAGLN2 affected TME play in tumor development. The heatmap provided in **Figure 6A** showed the correlation grade between TAGLN2 expression and the various TME terms, wherein antigen processing machinery, DNA replication, base excision repair, nucleotide excision repair, mismatch excision repair, and DNA damage response had a strong positive correlation with TAGLN2 expression levels in most cancers.

To further validate the involvement of TAGLN2 in TME, we calculated the ESTIMATEScore, ImmuneScore, and StromalScore in 33 cancer types based on expression data profiles** (Figure 6B-D)**. The results of the ESTIMATE algorithm indicated that TAGLN2 expression was positively correlated with ESTIMATEScore, ImmuneScore, and StromalScore in KICH, PRAD, UVM, LGG, SARC, THCA, PCPG, KIRC, DLBC, GBM, LIHC, KIRP, and BLCA. Yet remarkable inverse correlations were observed between TAGLN2 and ESTIMATEScore, ImmuneScore, and StromalScore in STAD and UCEC.

Furthermore, concerning Pearson's r, the five cancers with the most prominent positive correlations of TAGLN2 expression and TME-relevant scores were identified, for instance, KICH, PRAD, UVM, LGG, and SARC (**Figure S4A**, sorted by ESTIMATEScore); KICH, PRAD, UVM, LGG, and UCS (**Figure S4B**, sorted by ImmuneScore); KICH, LGG, PRAD, TGCT, and UVM (**Figure S4C**, sorted by StromalScore), respectively. Collectively, we postulated that TAGLN2 might be involved in remodeling the TME in certain types of cancer.

### The Relevance of TAGLN2 Expression and Immune Cell Infiltration in Pan-cancer

**Figure 7:**
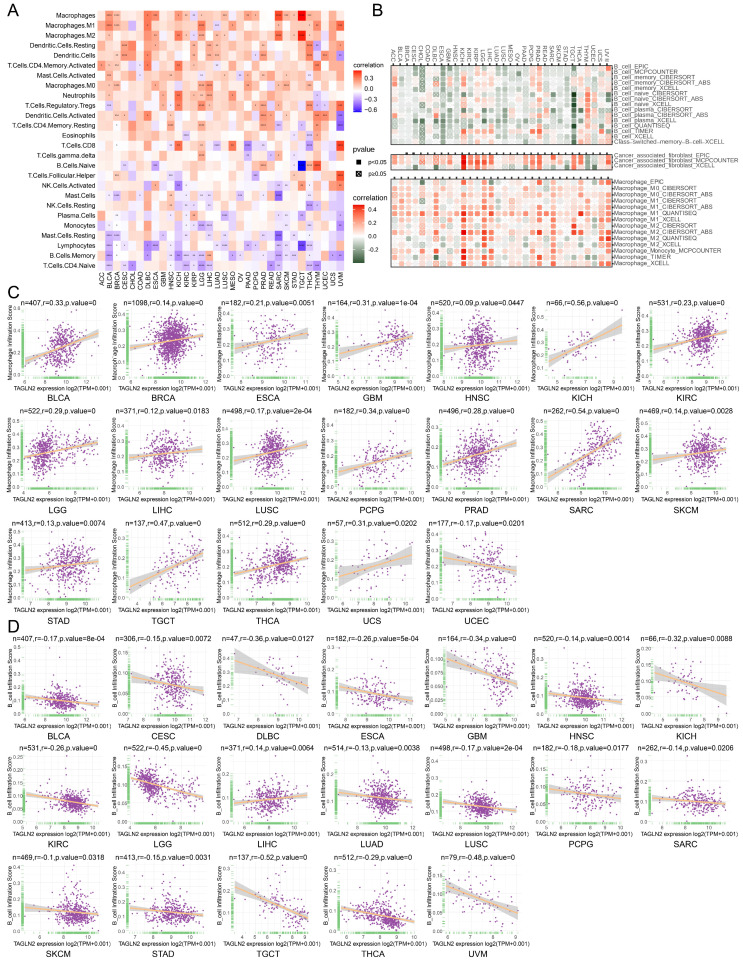
Relationship of TAGLN2 expression with immune cell infiltration analysis. (A) Heatmap depicting the relationship between TAGLN2 expression levels and the levels of infiltration of 26 immune-related cells. (B) Heatmap depicting the correlation between TAGLN2 expression and macrophages, cancer-associated fibroblasts, and B cells infiltration levels by using TIMER2.0 database. (C-D) Scatter plots displaying the pearson correlation between TAGLN2 expression and macrophage infiltration score(C) and B cell infiltration score(D) by using ImmuCellAI database. * p-value < 0.05, ** p-value < 0.01, *** p-value < 0.001, and **** p-value < 0.0001.

Tumor-infiltrating immune cells play a significant role in the promotion or inhibition of tumor growth, such as tumor-infiltrating B lymphocytes (TIBs), CD8^+^ T cells, and macrophages as integral components of the tumor microenvironment, exist in all stages of cancer and play important roles in shaping tumor development^25^-^28^. Aiming to investigate the correlation between immune cell infiltration and TAGLN2 expression at the pan-cancer level, we explored various publicly available data repositories to analyze the relationship between these two variables. According to the data published and evaluated by the “CIBERSORT” algorithm, TAGLN2 expression was intuitively found to be positively correlated with the infiltration levels of multiple immune cells, namely macrophages, dendritic cells, CD4^+^ T cells, and neutrophils. Conversely, it was observed to be negatively correlated with B cells, naïve T cells, and NK cells **(Figure 7A)**. Furthermore, an analysis of the potential relationship between TAGLN2 expression and the infiltrating levels of different immune cells in various cancer types was conducted using the TIMER2.0 portal. As expected, a significant positive correlation between TAGLN2 and macrophages infiltrating, as well as a negative association between TAGLN2 and the infiltration level of B cells was observed **(Figure 7B)**. The full analysis results can be found in **Figure S8**. Notably, we also proved a positive correlation between cancer-associated fibroblasts and TAGLN2 expression in most cancer types, excluding THYM and UCEC. Consistent with the above findings, the data obtained from the ImmuCellAI database revealed the same results, displaying a positive correlation between TAGLN2 expression and the level of macrophages in pan-cancer **(Figure 7C)**, while a converse association was observed with B cells** (Figure 7D)**. In short, these results suggested that TAGLN2 may be implicated in its tumorigenic role in most tumor types by regulating the infiltration levels of immune cells such as macrophages and B cells.

### Association of TAGLN2 With Immune-related Genes Across Multiple Cancers

**Figure 8:**
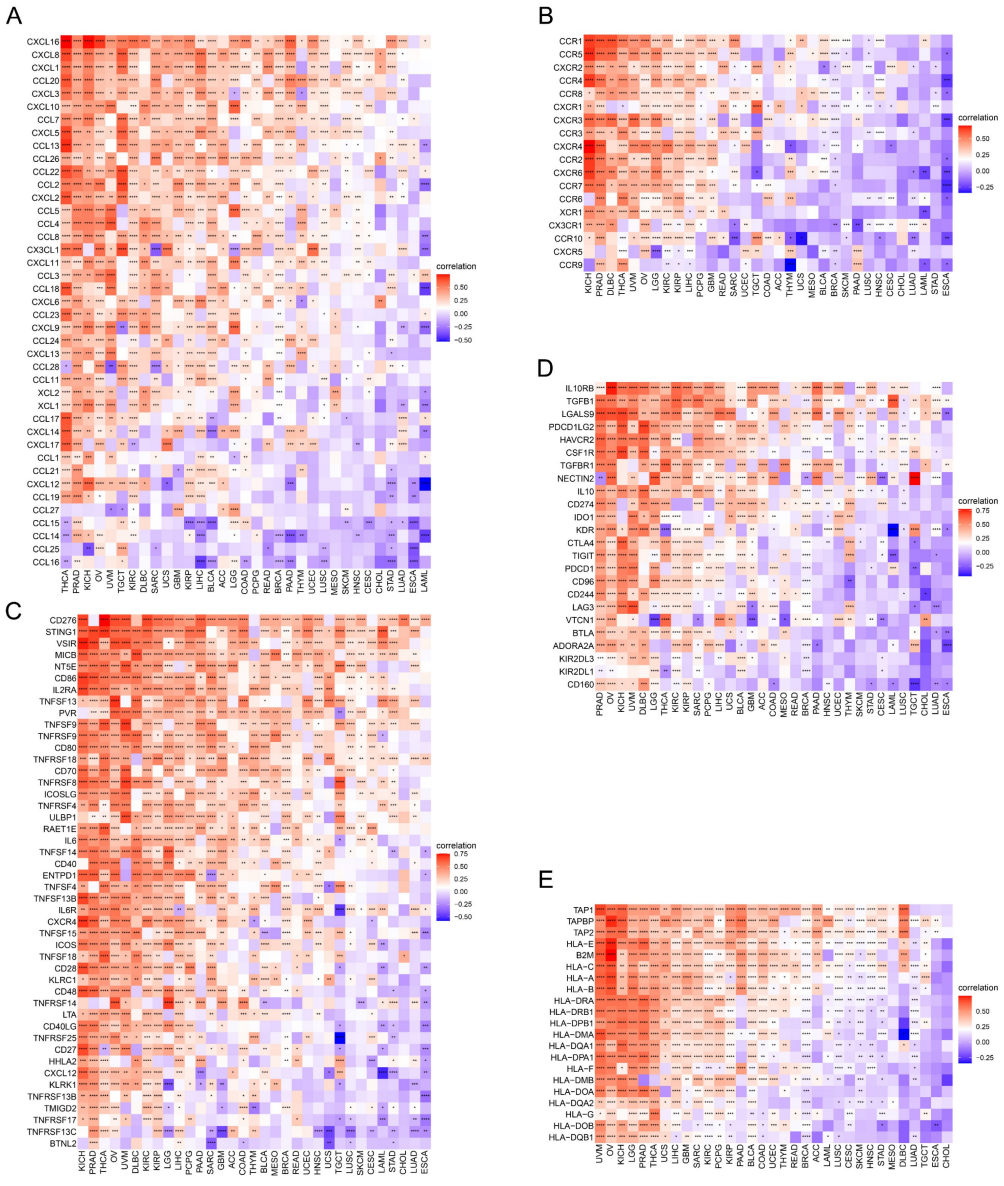
Relationship of TAGLN2 expression and immunoregulators. Co-expression of TAGLN2 and 150 immune moderator genes of five immune pathways, including chemokines(A), chemokine receptors(B), immune-activating genes(C), immunosuppressive genes(D), and MHC genes(E). * p-value < 0.05, ** p-value < 0.01, *** p-value < 0.001, and **** p-value < 0.0001.

Succeeding, the correlations of expression levels between TAGLN2 and immune-related genes that encode chemokines** (Figure 8A)**, chemokine receptors** (Figure 8B)**, immune-activating genes** (Figure 8C)**, immunosuppressive genes** (Figure 8D)**, and MHC genes** (Figure 8E)** were probed across different cancers. As evidenced by the findings, it can be clearly established that almost all immune-related genes were co-expressed with TAGLN2, with the omission of CHOL. Specifically, chemokine receptors such as CCR1, CCR5, and CXCR2, as well as chemokines such as CXCL16, CXCL8, CXCL1, CCL20, and CXCL3, were found to be strongly positively correlated with TAGLN2 expression, particularly in THCA, PRAD, KICH, OV, and UVM. In KICH, PRAD, OV, UVM, and DLBC, the heatmap exhibited a close positive relationship between TAGLN2 expression and immune-activating and immunosuppressive genes. In addition, we also itemized the correlations between TAGLN2 and the major immune checkpoints in pan-cancer, including LAG3, PDCD1, CTLA4, CD274, and TIGIT **(Figure S5)**. Moreover, a high degree of correlation between TAGLN2 and MHC genes was observed in most tumor types. Taken together, the outcomes of these studies showed that TAGLN2 was deeply involved in tumor immunity.

### The Relationships Between TAGLN2 Expression and TMB and MSI

**Figure 9:**
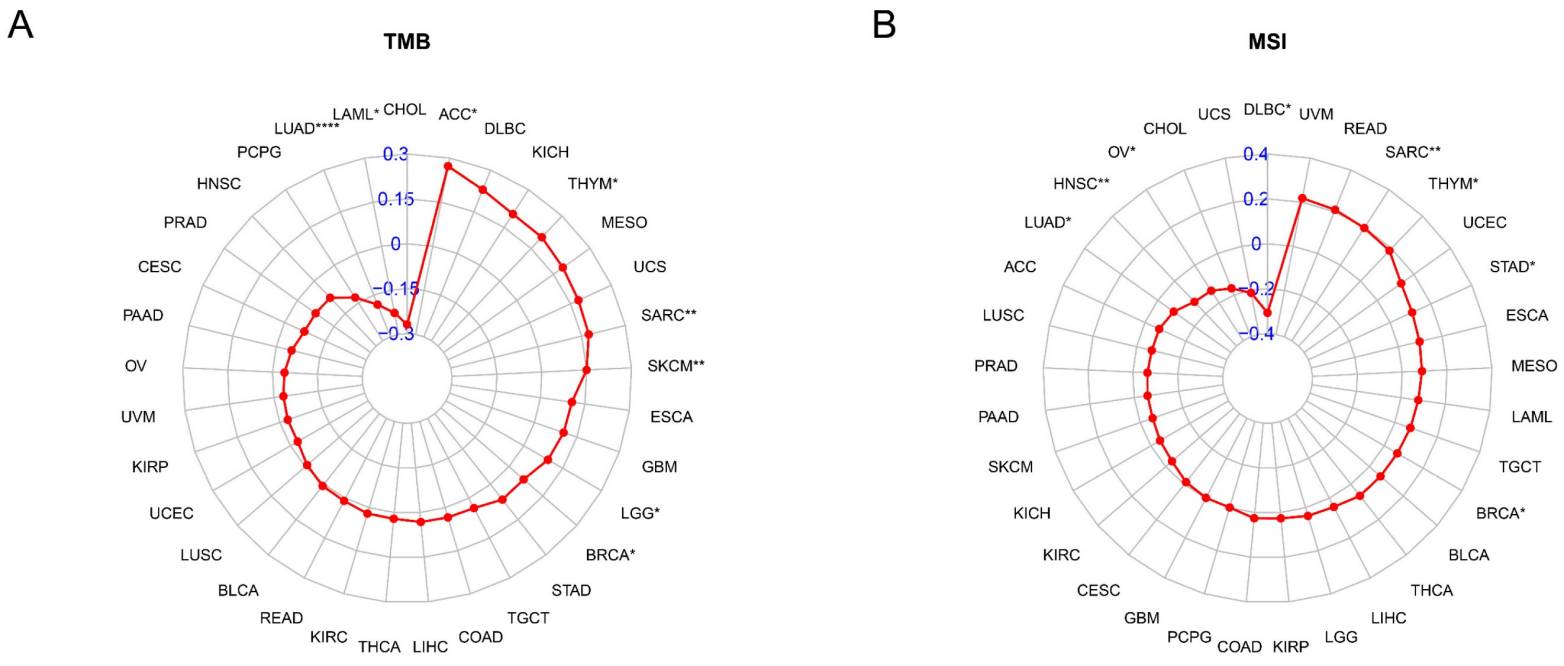
The correlation between TAGLN2 expression and TMB, and MSI in cancers. (A) Rader map illustrating the correlations between TAGLN2 expression and TMB. (B) Rader map illustrating the correlations between TAGLN2 expression and MSI. * p-value < 0.05, ** p-value < 0.01, *** p-value < 0.001, and **** p-value < 0.0001.

The TMB and MSI are known to contribute to the neoantigen load in tumors, thereby promoting the infiltration of immune effector cells, which has become a major predictive marker for the effectiveness of immune checkpoint blockade in recent years. Thus, we evaluated the connection between TAGLN2 and TMB, as well as MSI across 33 cancer types and depicted the data in the form of Rader graphs and scatter plotters. Accurately, a statistically significant positive correlation between TAGLN2 expression and TMB was observed in 6 cancer types, including ACC, THYM, SARC, SKCM, LGG, and BRCA, but TAGLN2 was negatively correlated to TMB in LUAD, and LAML **(Figure 9A)**. Furthermore, significant correlations between the expression of TAGLN2 and MSI were found in 8 cancer types, namely SARC, THYM, STAD, BRCA, LUAD, HNSC, OV, and DLBC, in which LUAD, HNSC, OV, and DLBC patients were negatively correlated to TAGLN2 expression, while the other cancer types showed the opposite trend **(Figure 9B)**. Scatter plots for each tumor mentioned were presented in **Figure S6A-D**.

### Functional Enrichment Analysis of TAGLN2 in Multiple Cancers

To investigate the potential functional mechanism of TAGLN2 in carcinogenesis, we carried out GSEA and GSVA analyses in pan-cancer subjects and the results of six tumors were presented in **Figure 11-13**.

**Figure 10:**
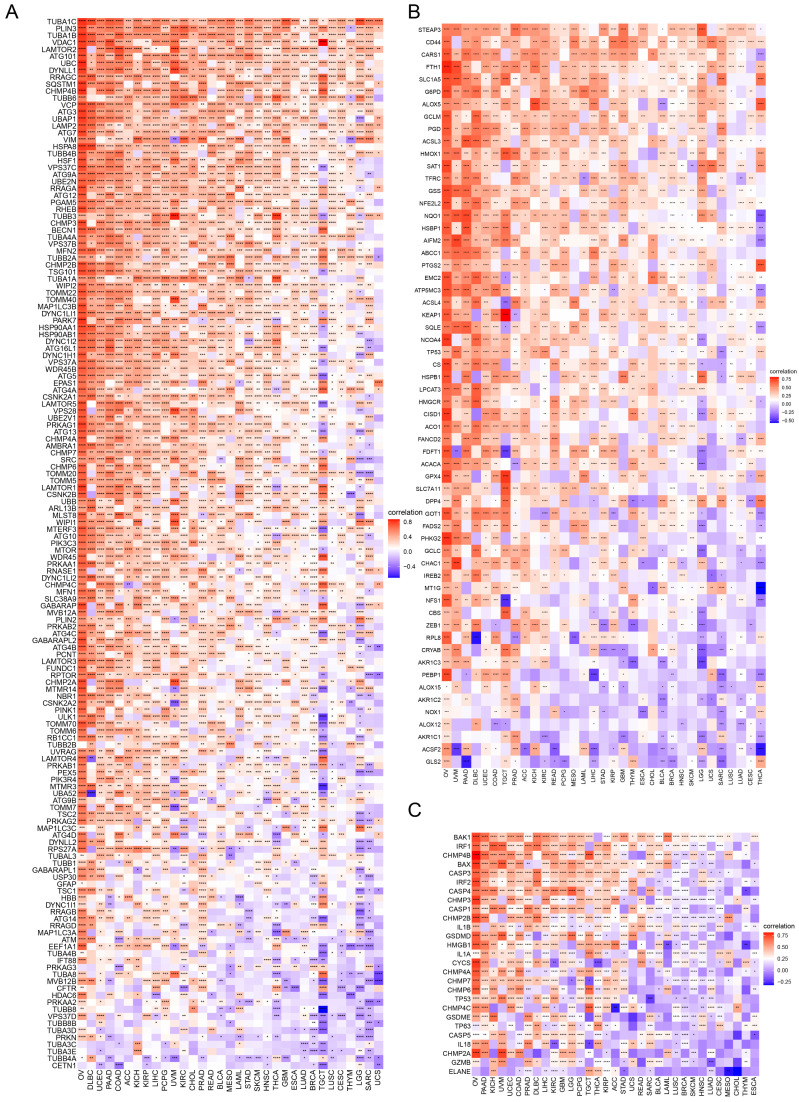
The correlation analysis between TAGLN2 and programmed cell death-related genes. (A-C) Co-expression of TAGLN2 and autophagy-related genes(A), ferroptosis-related genes(B), and pyroptosis-related genes(C). * p-value < 0.05, ** p-value < 0.01, *** p-value < 0.001, and **** p-value < 0.0001.

**Figure 11:**
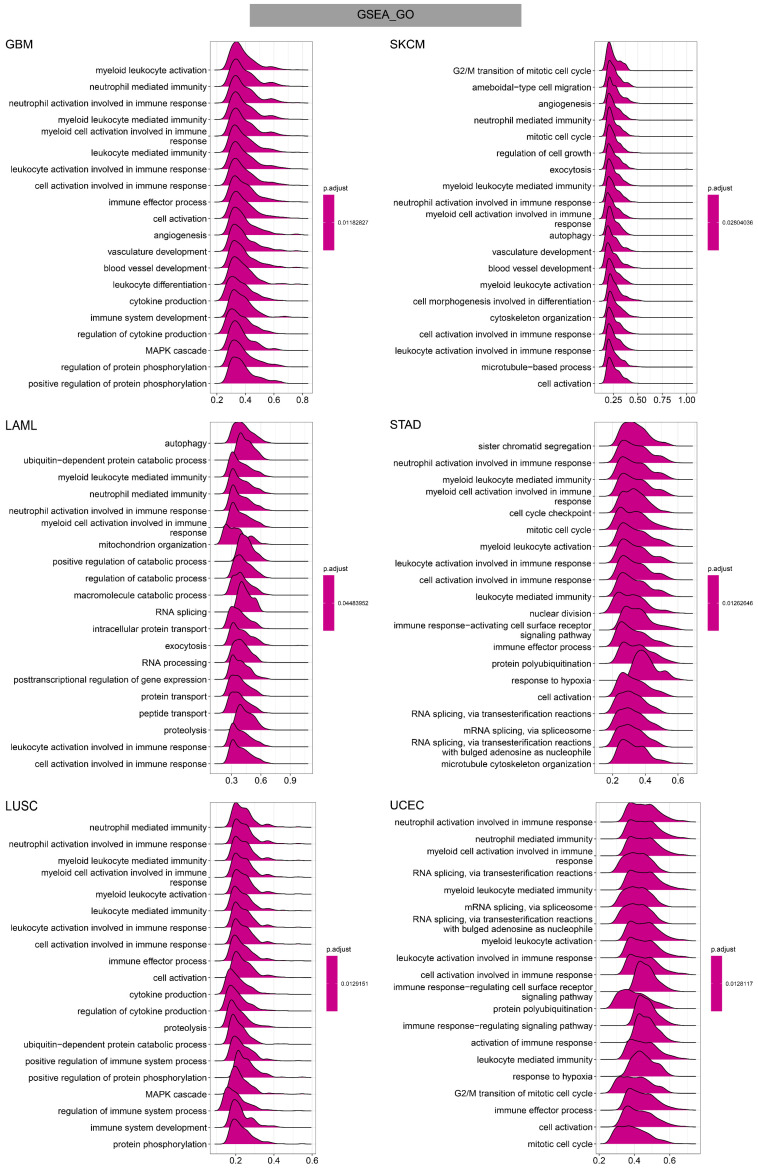
GO annotations of TAGLN2 in the indicated six types of tumors using GSEA.

**Figure 12:**
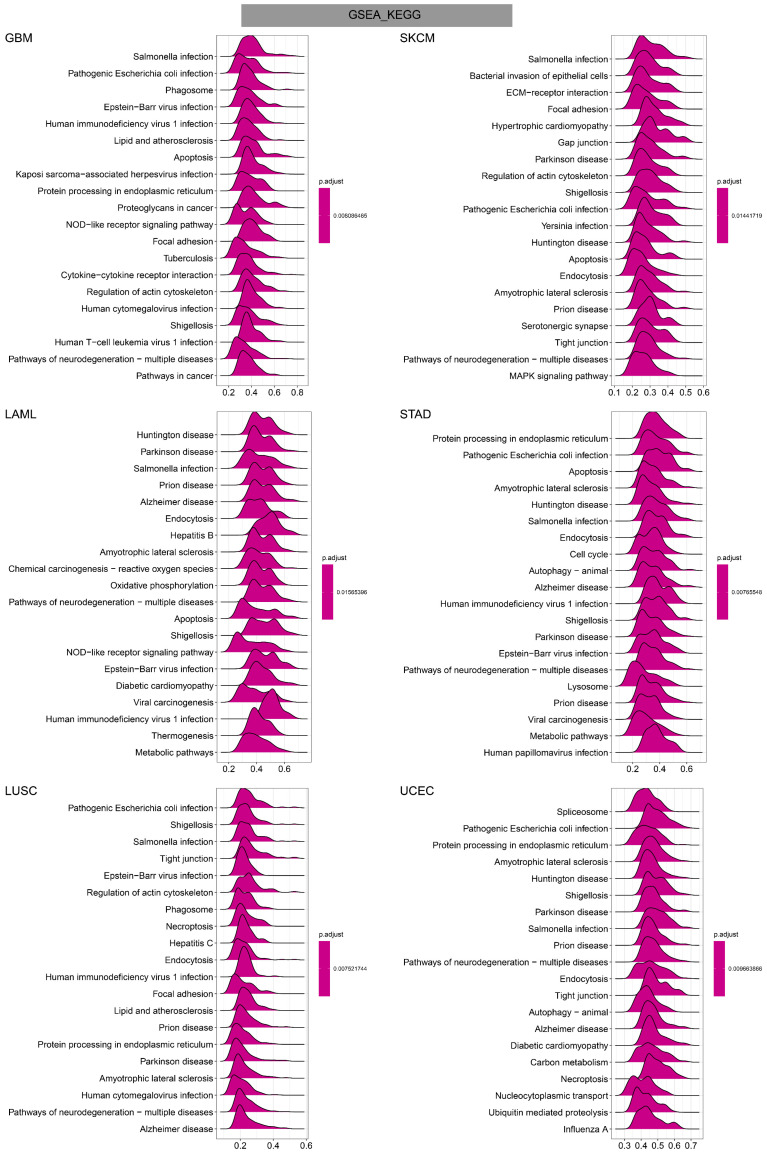
KEGG annotations of TAGLN2 in the indicated six types of tumors using GSEA.

In GO terms, we revealed that TAGLN2 mainly focused on the immune regulation-related mechanisms, especially for the neutrophil mediated immunity, immune effector process, and multiple cells activation involved in immune response. Besides, we noticed that TAGLN2 was closely linked to cell cycle or mitosis regulation in some certain cancer types **(Figure 11)**. Moreover, KEGG analysis suggested that the role of TAGLN2 in the pathogenesis of cancer may be related to programmed cell death (such as apoptosis, and necroptosis), epstein-barr virus infection, human immunodeficiency virus 1 infection, and cell junction (such as “Tight junction” and “Focal adhesion”), etc. **(Figure 12)**. Subsequent to the GSEA analysis, correlation heatmaps between TAGLN2 and other programmed cell death-related key genes (e.g. autophagy, ferroptosis, and pyroptosis) were further generated, which revealed a strong association between them in a variety of tumors **(Figure 10)**.

**Figure 13:**
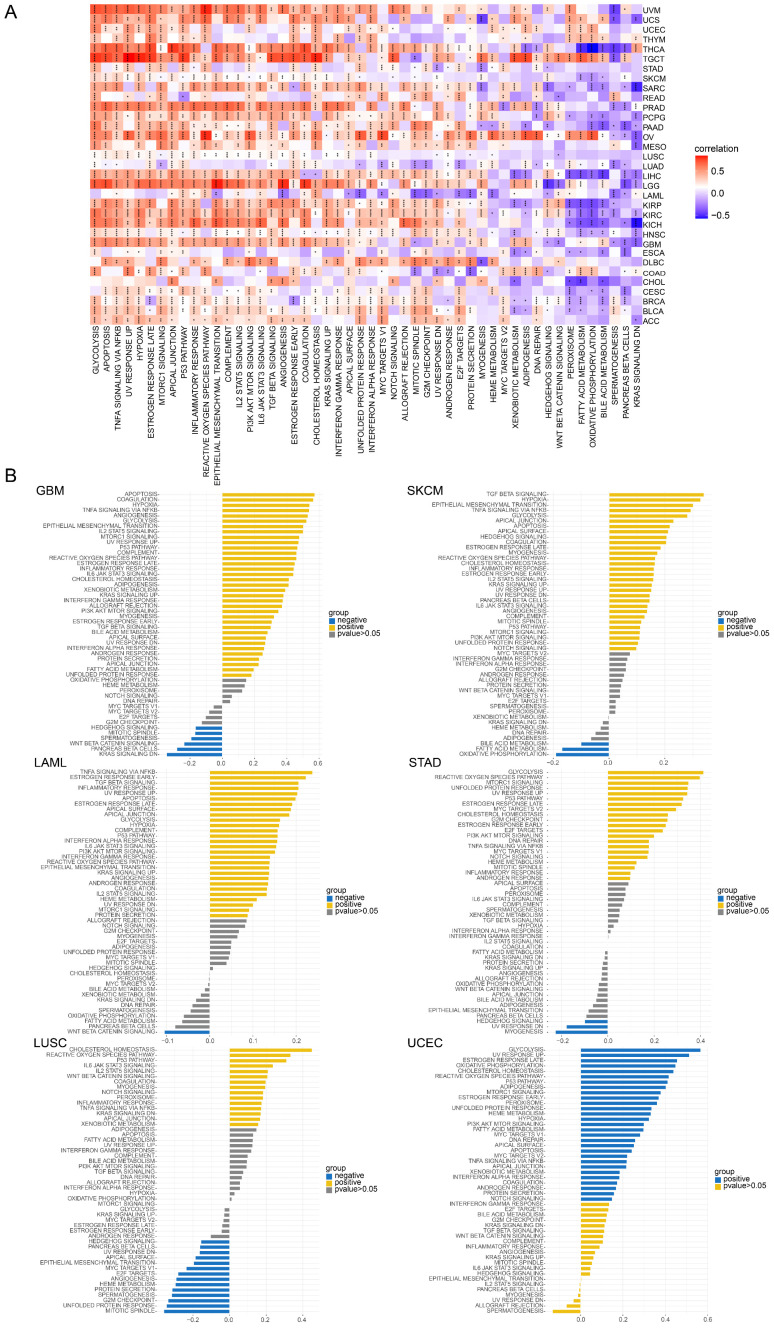
GSVA of TAGLN2 in pan-cancer. (A) Heatmap for different hallmark pathway enrichment scores with TAGLN2 expression level. (B) GSVA data of TAGLN2 in the indicated six types of cancers. * p-value < 0.05, ** p-value < 0.01, *** p-value < 0.001, and **** p-value < 0.0001.

Afterward, GSVA analysis of different hallmark pathways enrichment scores with TAGLN2 expression levels was displayed in the form of a heatmap from the MsigDB database** (Figure 13A)**. We amplified that TAGLN2 was positively correlated with most cancer-promoting pathways, such as glycolysis, apoptosis, hypoxia, EMT, PI3K AKT MTOR signaling, and angiogenesis. Likewise, the inflammatory response of cell immune factors, such as interferon-gamma response, interferon-alpha response, IL6, and TGF-beta, showed a strong positive correlation with TAGLN2 in most tumors. These were further corroborated by the results of **Figure 13B**. These results suggested the possibility that TAGLN2 regulated tumor progression from various perspectives, with the immunity and cell death/cycle-related aspects being the focus.

### Single-cell Functional Analysis of TAGLN2 Across Human Cancers

**Figure 14:**
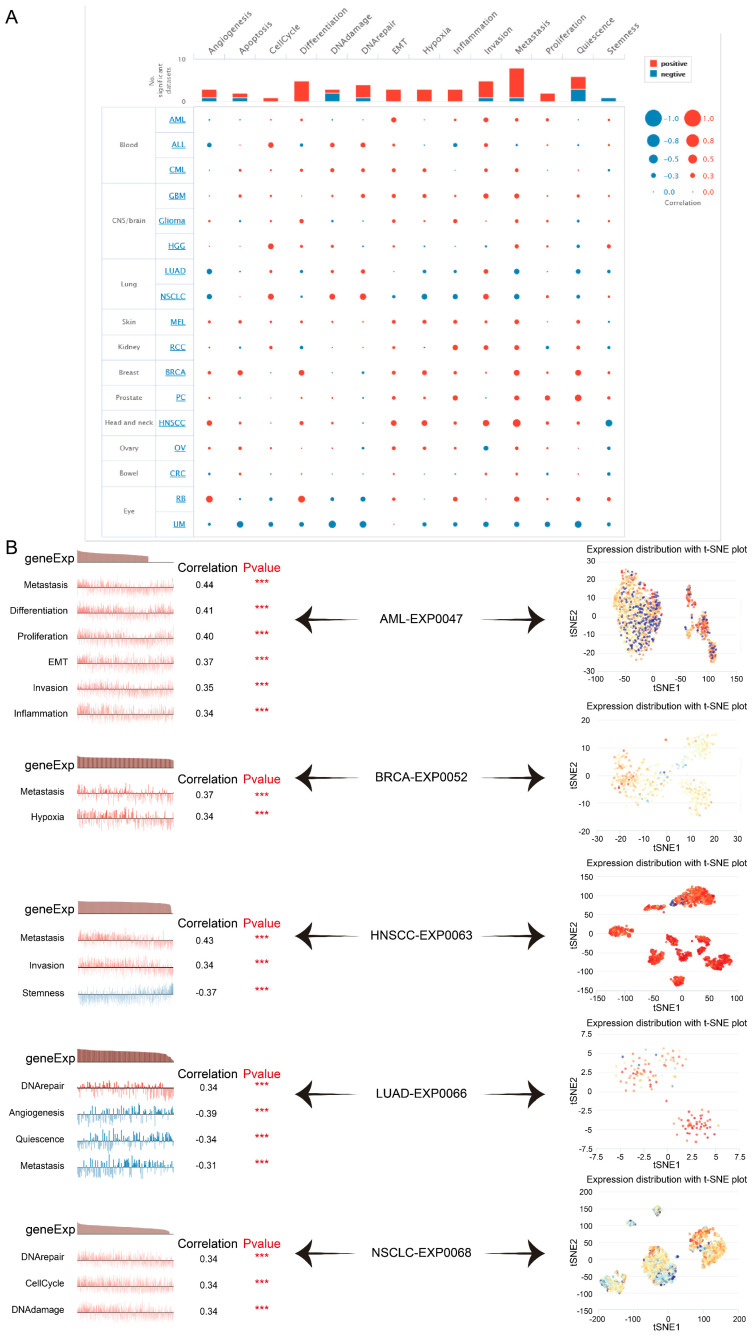
The tumor-related biological functional status of TAGLN2 at a single-cell level according to CancerSEA database. (A) Average correlations between TAGLN2 and functional states in different cancers. (B) The correlation between TAGLN2 expression and different tumor functional status in indicated datasets. (C) T-SNE diagram presenting TAGLN2 expression distribution of AML-EXP0047, BRCA-EXP0052, HNSCC-EXP0063, LUAD-EXP0066, and NSCLC-EXP0068 at a single-cell level. *** p-value < 0.001.

Here we verified that at single-cell resolution by mining the CancerSEA database, there was positive relativity between TAGLN2 expression and functional states, including EMT, metastasis, invasion, DNA damage, DNA repair, and hypoxia in multiple cancers **(Figure 14A)**. After that, we itemized the correlation between TAGLN2 and the various functional status in individual datasets **(Figure 14B)**. In agreement with the aforementioned results, TAGLN2 positively correlated with metastasis in AML-EXP0047, BRCA-EXP0052, and HNSCC-EXP0063 single-cell datasets; with DNA repair in LUAD-EXP0066, and NSCLC-EXP0068 single-cell datasets. Moreover, the t-SNE diagram illustrated the TAGLN2 expression profiles on a single cell basis within these datasets** (Figure 14C)**.

### Study on Correlation Between Drug Sensitivity (IC50) and TAGLN2 Expression

**Figure 15:**
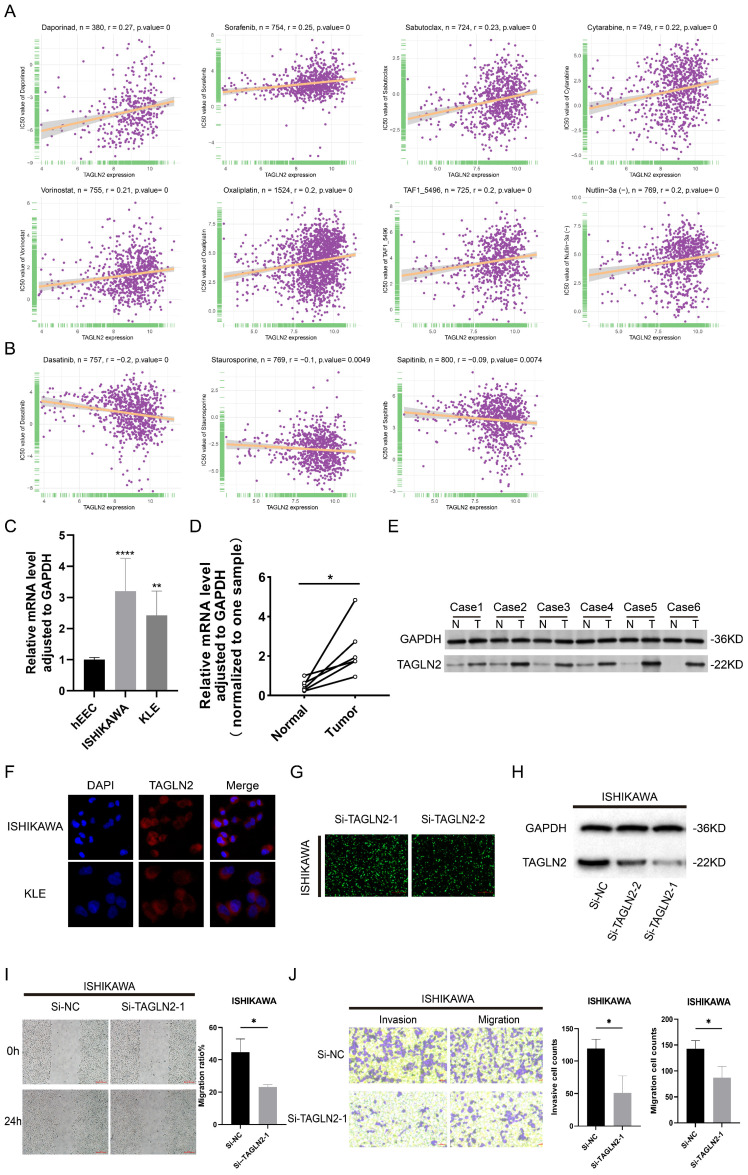
The correlation between drug sensitivity and TAGLN2 expression levels, as well as the validation of TAGLN2 effects on UCEC. (A) The top 8 with the positively highest spearman correlation scores. (B) The only 3 with the negatively highest spearman correlation scores. (C, D) TAGLN2 mRNA expression in UCEC cell lines(C) and paired tissues(D). (E) Western blot validation of TAGLN2 protein expression in UCEC paired tissues. (F) Protein distribution of TAGLN2 in ISHIKAWA and KLE cell lines. Blue fluorescence, DAPI, a specific dye staining DNA to localize the cell nucleus; red fluorescence, DyLight, combines with primary antibody, showing the distribution of target protein. (G-H) The fluorescence images(G), and western blot result(H) presenting the TAGLN2 knockdown efficiency of transfection in ISHIKAWA cells. (I) Wound healing assays in TAGLN2-knockdown ISHIKAWA cells following transfection with the Si-TAGLN2-1. (J) Transwell assays in TAGLN2-knockdown ISHIKAWA cells following transfection with the Si-TAGLN2-1, invasive cell numbers in the lower panel. * p-value < 0.05, ** p-value < 0.01, *** p-value < 0.001, and **** p-value < 0.0001.

Gene alterations strongly influence clinical responses to treatment and in many instances are potent biomarkers for response to drugs^29^. As a result, we incorporated large drug sensitivity and genomic datasets (GDSC database) to explore the association between mRNA expression levels of TAGLN2 and IC50 to antitumor drugs. We conducted correlation analysis for 192 drugs, and eventually, 130 drugs were proved to be correlated with TAGLN2, of which 127 were positively correlated and 3 were negatively correlated **(Table S1)**. Besides, there was no significant relevance between TAGLN2 and IC50 in a total of 62 compounds. We exhibited the top 8 drugs with the strongest positive correlations, including Daporinad, Sorafenib, Sabutoclax, Cytarabine, Vorinostat, Oxaliplatin, TAF1_5496, and Nutlin-3a(-) **(Figure 15A)**, together with the only 3 drugs with the negative correlations, namely Dasatinib, Staurosporine, and Sapitinib **(Figure 15B)**. The results of the complete drug sensitivity analysis were unveiled in **Table S1**. According to incomplete statistics, the commonly used anti-tumor drugs in the clinical treatment of carcinoma, such as 5-fluorouracil, cisplatin, gemcitabine, paclitaxel, crizotinib, oxaliplatin, niraparib, and tamoxifen, had higher IC50 values (worse efficacy) in patients with high TAGLN2 expression **(Figure S7)**. Overall, these results hinted that the expression level of TAGLN2 had an impact on the sensitivity of anti-tumor drugs.

### TAGLN2 was highly expressed and influenced the migration, and invasion of UCEC cells

According to the aforestated analysis results, we recognized the potential role of TAGLN2 in pan-cancer, especially in UCEC. Therefore, we selected UCEC as a representative type, with in vitro experiments to further validate the expression pattern and biological function of TAGLN2 in UCEC. In line with the aforestated analysis results, our result in **Figure 15F** demonstrated that the TAGLN2 protein was distributed in the whole cells and the abundance in the cytoplasm by immunofluorescence experiments. Subsequently, we confirmed that TAGLN2 was highly expressed in UCEC cells, including ISHIKAWA, and KLE, when compared to the normal human uterine endometrial epithelium cell line (hEEC) **(Figure 15C).** The result of qRT-PCR and western blot demonstrated that TAGLN2 was remarkably upregulated in UCEC tissues with the peritumoral tissues **(Figure 15D-E)**. Next, according to the expression level of TAGLN2 in 2 UCEC cell lines, we chose the cell line with the highest endogenous expression, ISHIKAWA, to conduct knockdown analysis and subsequent cell functional experiments.

As evidenced in **Figure 15G-H**, the results of fluorescence microscopy and western blot indicated Si-TAGLN2-1 had more excellent efficiency in the knockdown of the TAGLN2 expression. By performing wound healing and transwell assays, the migration and invasion capacities were suppressed of the si-RNA-transfected cells **(Figure 15I-J)**. All in all, consistent with previous studies, in UCEC, TAGLN2 was upregulated in cell lines and tumor tissues and led to a pro-tumorigenic effect.

## Discussion

Cancer is a serious and life-threatening disease that affects millions of people around the world. In recent years, advancements have been made in the treatment of cancer, however, the current treatments for cancer are not always effective. By exploring the genetic, epigenetic, and other molecular components that cause cancer, such as identifying specific genes or proteins involved in the cancer process, novel candidates for improved cancer treatments can be uncovered.

TAGLN2, located on the human chromosome 1q23.2 region, is a member of the transgelins family and encodes a 199 amino acid protein. Studies on TAGLN2 in the context of tumor progression have garnered considerable interest due to its ability to modulate the tumor microenvironment and promote tumor angiogenesis^30^, ^31^, invasion, and metastasis^32^, ^33^. Nevertheless, the exact mechanism of TAGLN2 in cancers remains largely unknown and research into it is still in its early stages. Thus, on the premise of the available public platforms, we operated a pan-cancer analysis, which provided valuable insights into the impact of TAGLN2 on multiple cancer types.

We first monitored in detail the expression levels of TAGLN2 mRNA and protein in human organs, tissues, and cell lines and compared them with those found in various cancers. Our findings revealed that TAGLN2 was extensively upregulated in 23 cancers, while presenting lower expression in KICH, LAML, PRAD, and THYM as compared to adjacent and normal tissues. The tumor cell lines expression levels from the CCLE confirmed the TAGLN2 mRNA expression discrepancy. Our results were further reinforced by the IHC analysis from HPA. These results were in accordance with previous reports regarding colorectal cancer^8^, gastric cancer^30^, bladder cancer^34^, clear cell renal cell carcinoma^35^, glioma^36^, and breast cancer^37^. Meanwhile, in patients having BLCA, KICH, KIRC, LIHC, LUSC, PAAD, TGCT, and THCA, TAGLN2 expression was analyzed to be correlated with the advanced cancer stage. Together, these results indicated that TAGLN2 might serve as an oncogene for tumor development and progression.

Later on, we evaluated the prognostic traits of TAGLN2 in 33 types of cancers which was a meaningful study to facilitate the translation of basic science to clinical studies. To get multifaceted results, four prognostic indexes (OS, DSS, DFI, and PFI) and two methods of statistics (Cox proportional hazard models and Kaplan-Meier survival curves) were used to analyze the data. In terms of OS, TAGLN2 was a hazard factor in several cancers, including LGG, UVM, LAML, LIHC, MESO, KICH, and ACC, and was correlated with shorter survival times. Simultaneously, high TAGLN2 showed as a risk factor in PAAD, KIRC, and LIHC, for DFI, DSS, and PFI. Existing evidence also suggested that TAGLN2 can be used as a single indicator to explain the survival probability of patients with KIRC^35^.

Likewise, Zhou et, al. proposed TAGLN2 was a novel target gene for the diagnosis, treatment, and prognosis of cervical cancer^38^. According to a published report, patients with high TAGLN2 mRNA expression were correlated with unfavorable overall survival, especially in estrogen receptor(ER)-negative breast cancer patients^37^. Additionally, a new 4-gene molecular marker that incorporated TAGLN2 was identified as an independent predictor of prognosis in patients with diffuse gliomas^39^. These results implied that elevated TAGLN2 expression may be related to increased mortality in these cancers and TAGLN2 tends to be an excellent prognostic value of several cancer types.

Gene alterations play a prominent role in driving cancer initiation and progression^40^. Initially, we displayed the genetic alteration landscape of TAGLN2 across all cancer types in the TCGA cohort, depicting that the most frequent alteration was amplification. And correlation analysis of CNVs levels of TAGLN2 and TAGLN2 expression levels showed a significant association between the two in 26 cancers. Huang et, al. clarified the transcriptional deregulation of TAGLN2 could be ascribed partially to their genomic aberrations in human hepatocellular carcinoma^41^. Of note, although TAGLN2 expression has been clarified in several cancers hitherto, its related research about TAGLN2 gene alteration in human cancers should be deeply explored in the future.

Epigenetic deregulation is a hallmark of cancer, and there has been increasing interest in therapeutics that target chromatin-modifying enzymes and other epigenetic regulators^42^. As one of the most abundant and well-studied epigenetic modifications, DNA methylation plays an essential role in normal development and cellular biology. Global alterations to the DNA methylation landscape contribute to alterations in the transcriptome and deregulation of cellular pathways^43^. Our study revealed that TAGLN2 expression was significantly inversely correlated with DNA methylation in various malignancies. Zhang et al. unearthed that lysine-40 succinylation of TAGLN2 induces glioma angiogenesis and tumor growth through regulating TMSB4X^31^.

Following that, promoter hypermethylation in TAGLN2 was strongly associated with tumor prognosis. Among them, TAGLN2 promoter hypermethylation resulted in better OS in patients diagnosed as KIRC, LGG, LIHC, MESO, THYM, and UVM, whereas the opposite was true for HNSC patients. However, additional evidence is merited for the potential role of TAGLN2 DNA methylation in tumorigenesis, which certainly offers new ideas and directions for future studies. These observations led us to hypothesize that mutations and epigenetic status of TAGLN2 in the genome might be involved in tumorigenesis.

TME is an integral part of cancer. Recognition of the essential nature of the TME in cancer evolution has led to a shift from a tumor cell-centered view of cancer development to the concept of a complex tumor ecosystem that supports tumor growth and metastatic dissemination^44^. The interaction of TME with cancer cells is responsible for tumor development, progression and drug resistance^45^.

The present research findings demonstrated that TAGLN2 expression in 33 malignancies had a positive correlation with TME involving antigen processing machinery, DNA damage, and repair; the latter two of these accounted for the majority. Interestingly, cancer cells are often characterized by abnormalities in DNA damage response including defects in DNA repair^46^. Deficiency in DNA damage response (DDR) genes leads to impaired DNA repair functions that will induce genomic instability and facilitate cancer development^47^. Consequently, TAGLN2 may influence DNA damage and repair and contribute to carcinogenesis. We calculated StromalScore and ImmuneScore to predict the level of infiltrating stromal and immune cells, which form the basis for the ESTIMATEScore to infer tumor purity in tumor tissue. And on the whole, higher StromalScore, ImmuneScore, and lower tumor purity were observed positive correlation with tumor stage and poor OS^48^. Concerning the ESTIMATEScore, ImmuneScore, and StromalScore, we observed that TAGLN2 had a considerably positive association in a variety of cancers, but only a negative association in STAD and UCEC when employing the TCGA cohort.

Immune cells, a key component of the TME, can protect us against tumor cells by inducing an anti-tumor immune response, such as tumor-infiltrated B cells that play an important role in humoral immunity^49^. On the other hand, they can also be pro-tumorigenic, as exemplified by tumor-associated macrophages that promote tumor progression through angiogenesis and lymphangiogenesis, immune suppression, hypoxia induction, tumor cell proliferation, and metastasis^25^, ^50^. In our investigation, we measured the quantity of immune cells infiltrating the 33 malignancies, which highlighted that TAGLN2 expression had a positive correlation with macrophages, dendritic cells, CD4^+^ T cells, and neutrophils and an inverse relationship with the infiltration level of B cells. Based on the positive correlation between TAGLN2 and tumor-associated macrophages, we can speculate that the tumor-promoting effect of TAGLN2 is partially due to the immunosuppressive phenotype of tumor-associated macrophages. At the same time, we also found a positive correlation between TAGLN2 and cancer-associated fibroblasts in most tumors examined, cancer-associated fibroblasts within the TME have been shown to play several roles in the development of a tumor, including the secretion of growth factors, inflammatory ligands, and extracellular matrix proteins that stimulate cancer cell proliferation, therapy resistance, and immune exclusion^51^. Furthermore, our study also uncovered the co-expression correlation of TAGLN2 with immune-related genes, including chemokine, chemokine receptor, MHC, immunostimulatory, and immunosuppressive genes, and the outcomes altogether showed that TAGLN2 is broadly involved in cancer immunity.

TMB and MSI are approved for clinical use to predict response to immunotherapy with immune checkpoint inhibitors in cancer patients^52^. Studies have shown a sustained clinical response to immune checkpoint inhibitors with dramatic clinical improvement in patients with MSI-H^53^. MSI as a predictive factor for treatment outcome of gastroesophageal adenocarcinoma^54^. For most cancer histologies, an association between higher TMB and improved survival was observed^55^. The present study found that TAGLN2 had a remarkable correlation with TMB in 8 cancers and MSI in 8 cancers. To this, these results suggested that TAGLN2 seemed to be capable of guiding the treatment of cancer to a certain degree.

After constructing a robust link between TAGLN2 and TME, we continued to use the GSEA analysis and GSVA analysis to explore the regulatory mechanism of TAGLN2 in the context of pan-cancer. GO analysis displayed the role of TAGLN2 in immune regulation-related mechanisms. Supporting this, Jun's team declared that TAGLN2^-/-^ DCs exhibited significant defects in their abilities to home to draining lymph nodes(LNs) and to form optimal contacts with cognate CD4^+^ T cells to prime T cells, and these changes were associated with a failure to suppress tumor growth and metastasis of B16F10 melanoma cells in mice^10^. They also revealed a novel function of TAGLN2 in enhancing T cell activation by stabilizing the immunological synapse^56^. Besides, TAGLN2-deficient macrophages showed defective phagocytic functions of IgM- and IgG-coated sheep red blood cells as well as bacteria^12^. KEGG analysis and programmed cell death-related key genes analysis uncovered another major portion of the possible mechanisms through which TAGLN2 operates, namely related to programmed cell death, such as apoptosis, necroptosis, autophagy, ferroptosis, and pyroptosis. Over the past decades, cancer drug discovery has significantly benefited from the use of small-molecule compounds to target classical modalities of cell death such as apoptosis, while newly identified cell death pathways has also emerging their potential for cancer drug discovery in recent years^57^-^61^. GSVA analysis further supplemented the above results, demonstrating the correlation between TAGLN2 and glycolysis, apoptosis, hypoxia, EMT, PI3K AKT MTOR signaling, inflammatory response, and angiogenesis. The above results provided the theoretical basis for TAGLN2 as a therapeutic target from different angles, making it a promising strategy for cancer therapy.

Although high functional heterogeneity of cancer cells, single-cell sequencing technology provides an unprecedented opportunity to decipher diverse functional states of cancer cells at single-cell resolution^62^. We identified that, at single-cell resolution, a positive correlation existed between TAGLN2 expression and functional states, such as EMT, metastasis, invasion, DNA damage, DNA repair, and hypoxia, across multiple cancers.

In terms of drug sensitivity to TAGLN2, 127 out of 192 antitumor drugs, including many common drugs, were less effective in the TAGLN2 high-expression group. Consistently, the inhibition of TAGLN2 expression with small interfering RNA sensitizes a paclitaxel-resistant human breast cancer cell line (MCF-7/PTX) to paclitaxel^63^. Phenolic compounds such as paeonol and salvianolic acid A can reverse the paclitaxel resistance in MCF-7/PTX cells as these compounds can both decrease TAGLN2 expression^64^-^67^. In line with these findings, Liu et, al. demonstrated TAGLN2 promotes paclitaxel resistance and the migration and invasion of breast cancer by directly interacting with PTEN and activating the PI3K/Akt/GSK-3β pathway^68^. TAGLN2 may therefore be useful as a novel therapeutic target to reverse the failure of treatments for diverse malignancies to some extent.

At last, we tested TAGLN2 expression and function in UCEC, which is a gynecological malignant tumor with a low survival rate and poor prognosis^69^. Meanwhile, the function of TAGLN2 in UCEC has not been validated, hence we selected UCEC to conduct further verification. Initially, we tested TAGLN2 distribution in cancer cells, and in line with our results, we unraveled that TAGLN2 was indeed abundant in the cytoplasm. TAGLN2 was also reported to be localized in the cytoplasm in Xiao's research^70^. And in accordance with our findings, we discovered that TAGLN2 was indeed upregulated both in UCEC cell lines and tumor tissues. More importantly, blocking TAGLN2 could significantly suppress the migration, and invasion of UCEC cells, suggesting that TAGLN2 may exert an oncogenic function in UCEC. However, the functional roles and specific molecular mechanisms of TAGLN2 need to be further clarified in UCEC.

Overall, our study is the first comprehensive analysis of TAGLN2 at pan-cancer. The findings presented in this study highlight the potential of TAGLN2 as a valuable biomarker for prognosis and immunotherapy and offer important implications for the development of novel therapies. Of note, we provide several unique perspectives on the potential use of TAGLN2 as a therapeutic target for cancer treatment. Firstly, our results reveal a potential role for TAGLN2 in the regulation of diverse subroutines of programmed cell death, including apoptosis, necroptosis, autophagy, ferroptosis, and pyroptosis, which have recently been reported to play significant roles in the modulation of cancer progression and are considered a promising strategy for cancer treatment. Secondly, we suggest a strong correlation between TAGLN2 expression and the composition and specific function of the tumor microenvironment, which is preponderant in regulating tumor progression and modulating response to standard-of-care therapies. Thirdly, emerging researches have established that the phosphorylation and succinylation of TAGLN2 exerts a pivotal function during the onset and progression of tumors. Our research, however, focuses on a distinct yet significant epigenetic modification - methylation - and presents compelling evidence that methylation of TAGLN2 is closely associated with the prognosis of certain types of cancer. Owing to its selectivity and specificity, targeted chemotherapy has emerged as a leading approach for cancer treatment in recent years, which allow for more effective treatment with fewer side effects. We aim to provide a more diverse and solid theoretical basis for utilizing TAGLN2 as a targeted molecular through our study. Furthermore, it is now clear that the TAGLN2 status of the cancer cell has a profound involvement on the cancer immunity. Another important contribution of our study is that it complements and enriches this conclusion from various facets, such as immune cell infiltration, immune-related genes, functional enrichment pathways, TMB, MSI, and major immune checkpoints. Nevertheless, there are a few shortcomings in the current research, such as the data utilized may be influenced by a systematic bias. The sample size of clinical tissues is relatively small. And the mechanism of TAGLN2 in cancer was mainly from bioinformatics analysis without laboratory confirmation. Consequently, we are keen to further investigate more functions, mechanisms, and potential therapeutic objectives of TAGLN2 in diverse cancers by designing a range of in vitro and in vivo experiments, and finally incorporating them with clinical practice.

## Conclusions

Collectively, we preliminarily illustrated the oncogenic role and prognostic value of TAGLN2 in pan-cancer. Further, TAGLN2 expression was associated with gene methylation, TME, infiltration of immune cells, immune-related genes, PCD, MSI, TMB, and drug sensitivity. These discoveries augmented the understanding of the functions of TAGLN2 in tumor growth and advancement, providing inspiration for clinical applications of TAGLN2-targeted therapies down the line.

## Supplementary Material

Supplementary figures and tables.

## Abbreviations

ACC: adrenocortical carcinoma
BLCA: bladder urothelial carcinoma
BRCA: breast invasive carcinoma
CESC: cervical squamous cell carcinoma and endocervical adenocarcinoma
CHOL: cholangiocarcinoma
COAD: colon adenocarcinoma
DLBC: lymphoid neoplasm diffuse large B-cell lymphoma
ESCA: esophageal carcinoma
GBM: glioblastoma
LGG: brain lower grade glioma
HNSC: head and neck squamous cell carcinoma
KICH: kidney chromophobe
KIRC: kidney renal clear cell carcinoma
KIRP: kidney renal papillary cell carcinoma
LAML: acute myeloid leukemia
LIHC: liver hepatocellular carcinoma
LUAD: lung adenocarcinoma
LUSC: lung squamous cell carcinoma
MESO: mesothelioma
OV: ovarian serous cystadenocarcinoma
PAAD: pancreatic adenocarcinoma
PCPG: pheochromocytoma and paraganglioma
PRAD: prostate adenocarcinoma
READ: rectum adenocarcinoma
SARC: sarcoma
SKCM: skin cutaneous melanoma
STAD: stomach adenocarcinoma
TGCT: testicular germ cell tumors
THCA: thyroid carcinoma
THYM: thymoma
UCEC: uterine corpus endometrial carcinoma
UCS: uterine carcinosarcoma
UVM: uveal melanoma
