# Supplementary material for: An Integrated Analysis Identified TAGLN2 As an Oncogene Indicator Related to Prognosis and Immunity in Pan-Cancer
Source: J Cancer. 2023 Jun 19;14(10):1809–36. doi: 10.7150/jca.84454 (PMC10355213; doi:10.7150/jca.84454)
Supplement: Supplementary file 1 — Supplementary figures and tables. [file jcav14p1809s1.pdf]

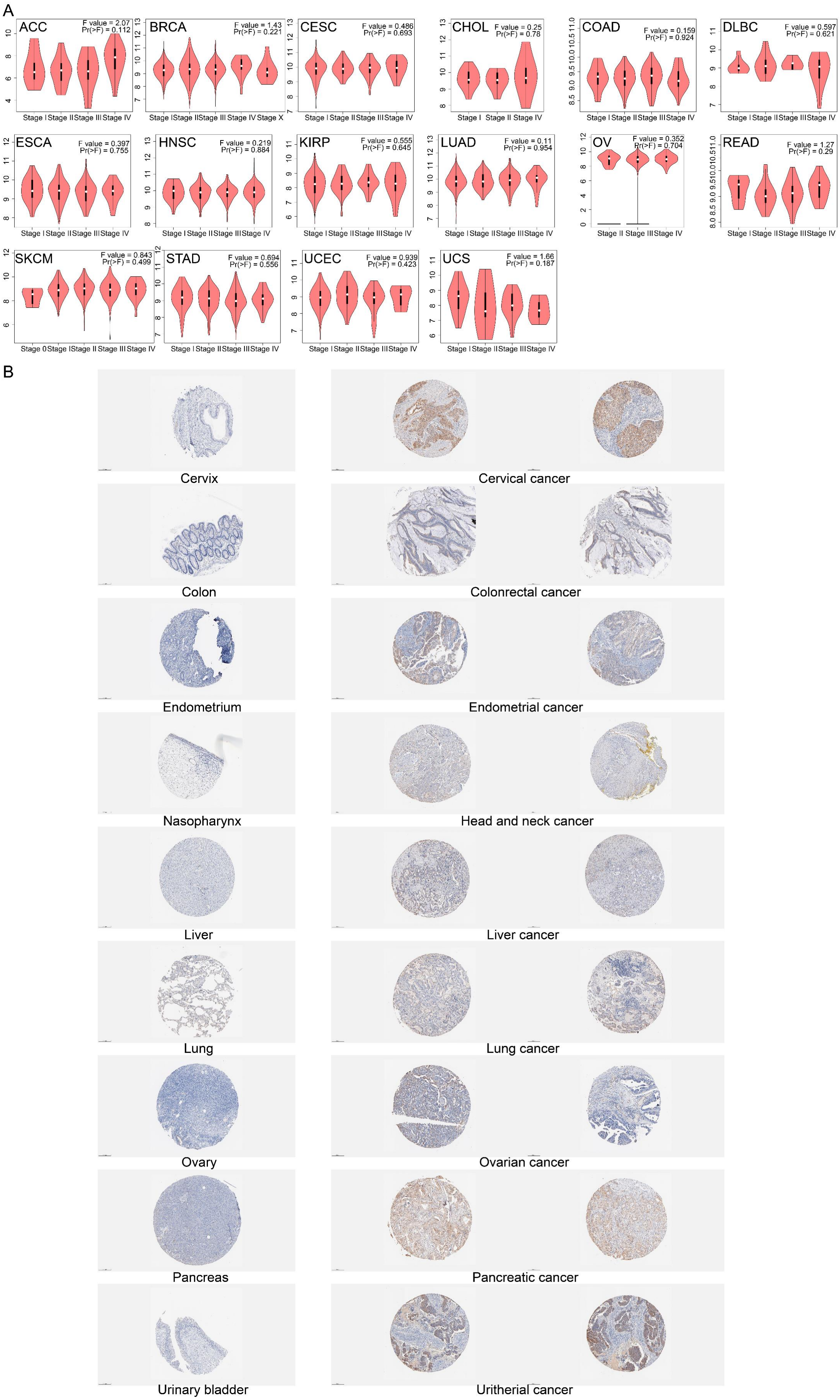
A
ACC
F value = 2.07
Pr(>F) = 0.112
BRCA
F value = 1.43
Pr(>F) = 0.221
CESC
F value = 0.486
Pr(>F) = 0.693
CHOL
F value = 0.25
Pr(>F) = 0.78
COAD
F value = 0.159
Pr(>F) = 0.924
DLBC
F value = 0.597
Pr(>F) = 0.621
ESCA
F value = 0.397
Pr(>F) = 0.755
HNSC
F value = 0.219
Pr(>F) = 0.884
KIRP
F value = 0.555
Pr(>F) = 0.645
LUAD
F value = 0.11
Pr(>F) = 0.954
OV
F value = 0.352
Pr(>F) = 0.704
READ
F value = 1.27
Pr(>F) = 0.29
SKCM
F value = 0.843
Pr(>F) = 0.499
STAD
F value = 0.694
Pr(>F) = 0.556
UCEC
F value = 0.939
Pr(>F) = 0.423
UCS
F value = 1.66
Pr(>F) = 0.187
B
Cervix
Cervical cancer
Colon
Colonrectal cancer
Endometrium
Endometrial cancer
Nasopharynx
Head and neck cancer
Liver
Liver cancer
Lung
Lung cancer
Ovary
Ovarian cancer
Pancreas
Pancreatic cancer
Urinary bladder
Uritherial cancer

Supplementary Figure 1. (A) The expression levels of TAGLN2 based on the different pathological stages analyzing using GEPIA2.0. (B) The immunohistochemistry images of TAGLN2 in different normal(left) and paired tumor(right) tissues from HPA database in indicated cancer types.

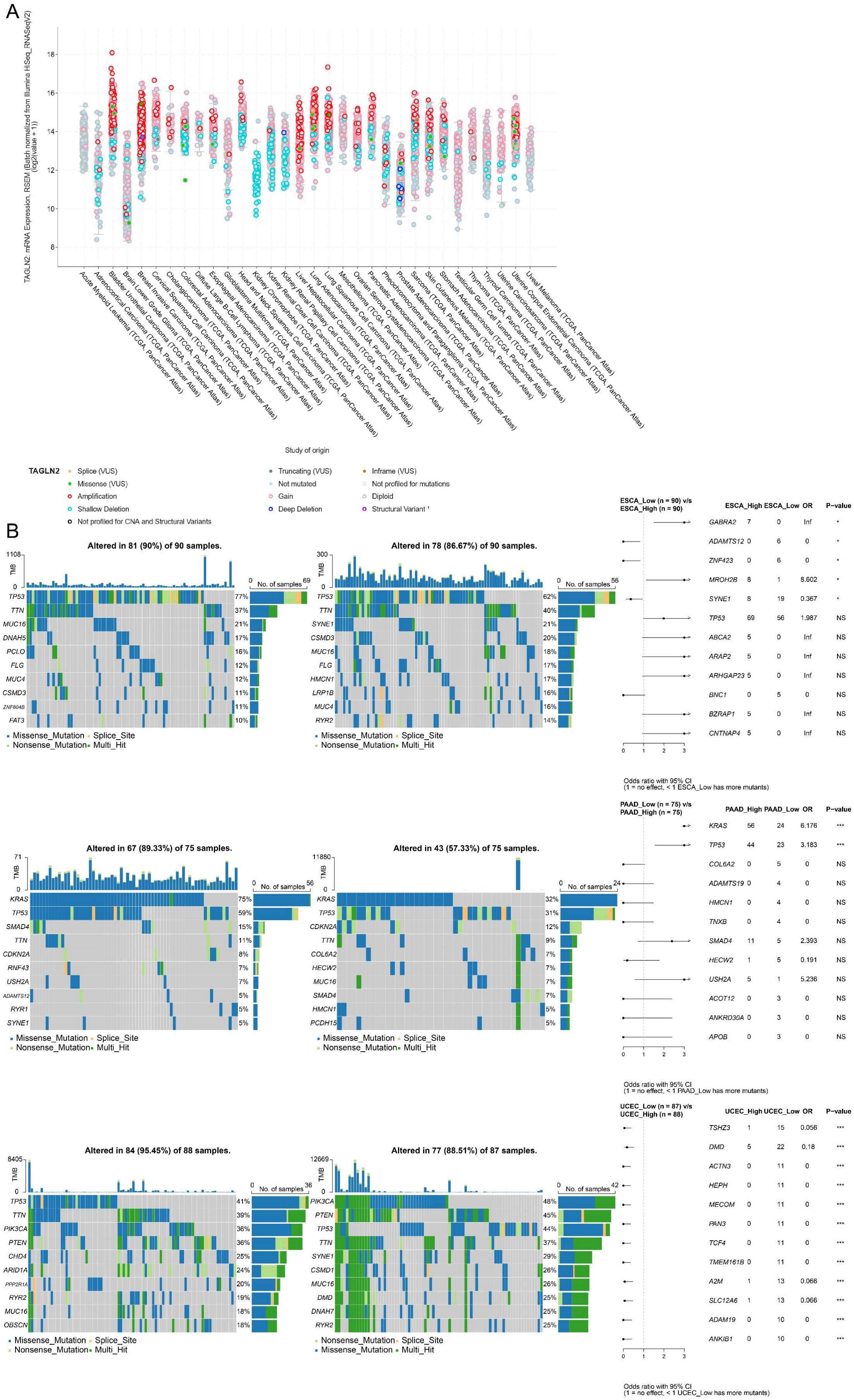
A
TAGLN2: mRNA Expression, RSEM (Batch normalized from Illumina HiSeq_RNASeqV2) (log2(value + 1))
Study of origin
TAGLN2
Splice (VUS)
Missense (VUS)
Amplification
Shallow Deletion
Not profiled for CNA and Structural Variants
Truncating (VUS)
Not mutated
Gain
Deep Deletion
Inframe (VUS)
Not profiled for mutations
Diploid
Structural Variant
B
Altered in 81 (90%) of 90 samples.
Altered in 78 (86.67%) of 90 samples.
ESCA_Low (n = 90) v/s ESCA_High (n = 90)
ESCA_High ESCA_Low OR P−value
GABRA2 7 0 Inf *
ADAMTS12 0 6 0 *
ZNF423 0 6 0 *
MROH2B 8 1 8.602 *
SYNE1 8 19 0.367 *
TP53 69 56 1.987 NS
ABCA2 5 0 Inf NS
ARAP2 5 0 Inf NS
ARHGAP23 5 0 Inf NS
BNC1 0 5 0 NS
BZRAP1 5 0 Inf NS
CNTNAP4 5 0 Inf NS
Odds ratio with 95% CI
(1 = no effect, < 1 ESCA_Low has more mutants)
Altered in 67 (89.33%) of 75 samples.
Altered in 43 (57.33%) of 75 samples.
PAAD_Low (n = 75) v/s PAAD_High (n = 75)
PAAD_High PAAD_Low OR P−value
KRAS 56 24 6.176 ***
TP53 44 23 3.183 ***
COL6A2 0 5 0 NS
ADAMTS19 0 4 0 NS
HMCN1 0 4 0 NS
TNXB 0 4 0 NS
SMAD4 11 5 2.393 NS
HECW2 1 5 0.191 NS
USH2A 5 1 5.236 NS
ACOT12 0 3 0 NS
ANKRD30A 0 3 0 NS
APOB 0 3 0 NS
Odds ratio with 95% CI
(1 = no effect, < 1 PAAD_Low has more mutants)
Altered in 84 (95.45%) of 88 samples.
Altered in 77 (88.51%) of 87 samples.
UCEC_Low (n = 87) v/s UCEC_High (n = 88)
UCEC_High UCEC_Low OR P−value
TSHZ3 1 15 0.056 ***
DMD 5 22 0.18 ***
ACTN3 0 11 0 ***
HEPH 0 11 0 ***
MECOM 0 11 0 ***
PAN3 0 11 0 ***
TCF4 0 11 0 ***
TMEM161B 0 11 0 ***
A2M 1 13 0.066 ***
SLC12A6 1 13 0.066 ***
ADAM19 0 10 0 ***
ANKIB1 0 10 0 ***
Odds ratio with 95% CI
(1 = no effect, < 1 UCEC_Low has more mutants)
Missense_Mutation
Splice_Site
Nonsense_Mutation
Multi_Hit

Supplementary Figure 2. (A) General mutation count of TAGLN2 in various TCGA cancer types according to the cBioPortal database. (B) Waterfall plots of tumor somatic mutation in the high- and low-risk groups in ESCA, PAAD, and UCEC. * p-value < 0.05, ** p-value < 0.01, *** p-value < 0.001, and **** p-value < 0.0001.

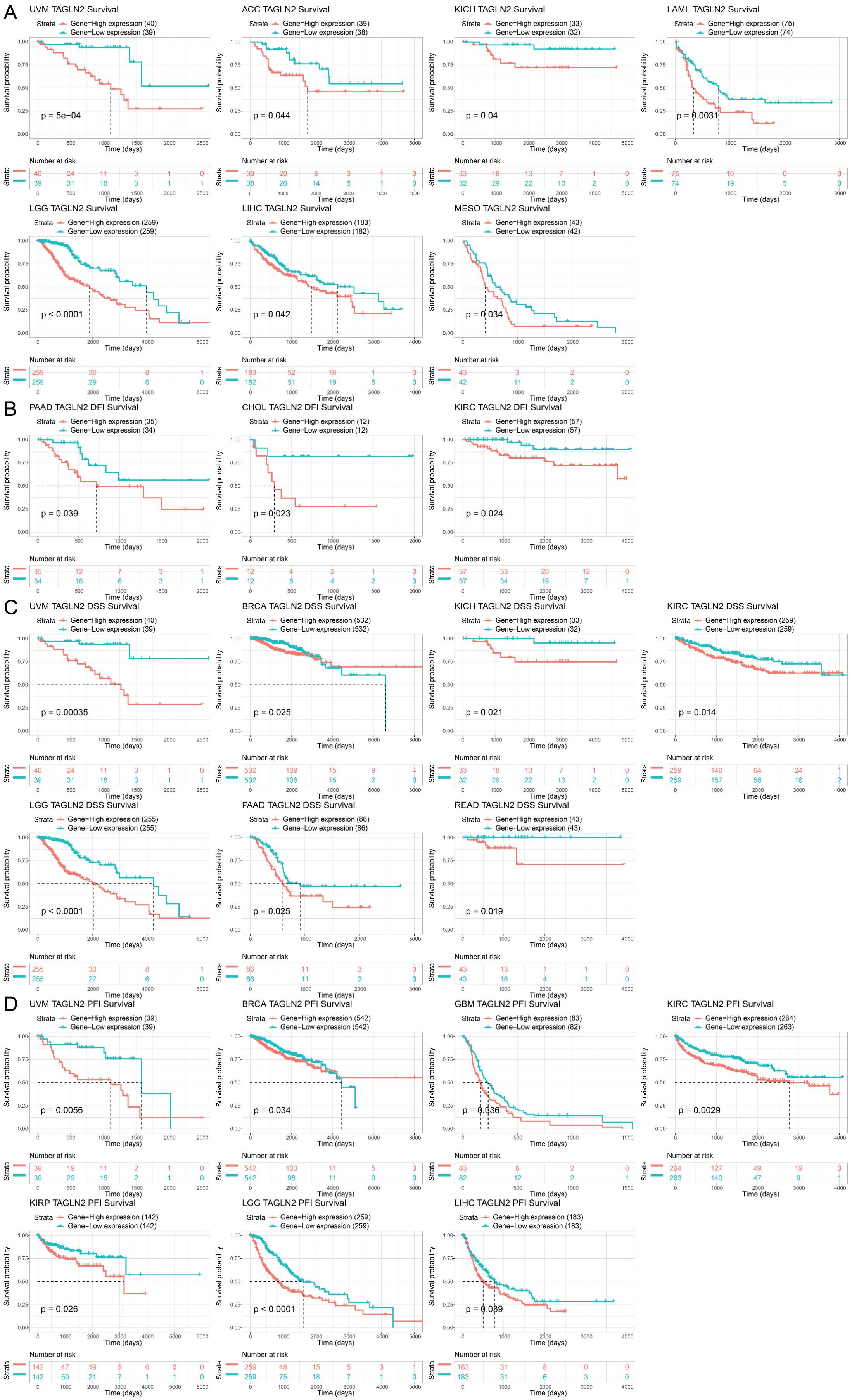
A
UVM TAGLN2 Survival
Gene=High expression (40)
Gene=Low expression (39)
p = 5e−04
ACC TAGLN2 Survival
Gene=High expression (39)
Gene=Low expression (38)
p = 0.044
KICH TAGLN2 Survival
Gene=High expression (33)
Gene=Low expression (32)
p = 0.04
LAML TAGLN2 Survival
Gene=High expression (75)
Gene=Low expression (74)
p = 0.0031
LGG TAGLN2 Survival
Gene=High expression (259)
Gene=Low expression (259)
p < 0.0001
LIHC TAGLN2 Survival
Gene=High expression (183)
Gene=Low expression (182)
p = 0.042
MESO TAGLN2 Survival
Gene=High expression (43)
Gene=Low expression (42)
p = 0.034
B
PAAD TAGLN2 DFI Survival
Gene=High expression (35)
Gene=Low expression (34)
p = 0.039
CHOL TAGLN2 DFI Survival
Gene=High expression (12)
Gene=Low expression (12)
p = 0.023
KIRC TAGLN2 DFI Survival
Gene=High expression (57)
Gene=Low expression (57)
p = 0.024
C
UVM TAGLN2 DSS Survival
Gene=High expression (40)
Gene=Low expression (39)
p = 0.00035
BRCA TAGLN2 DSS Survival
Gene=High expression (532)
Gene=Low expression (532)
p = 0.025
KICH TAGLN2 DSS Survival
Gene=High expression (33)
Gene=Low expression (32)
p = 0.021
KIRC TAGLN2 DSS Survival
Gene=High expression (259)
Gene=Low expression (259)
p = 0.014
LGG TAGLN2 DSS Survival
Gene=High expression (255)
Gene=Low expression (255)
p < 0.0001
PAAD TAGLN2 DSS Survival
Gene=High expression (86)
Gene=Low expression (86)
p = 0.025
READ TAGLN2 DSS Survival
Gene=High expression (43)
Gene=Low expression (43)
p = 0.019
D
UVM TAGLN2 PFI Survival
Gene=High expression (39)
Gene=Low expression (39)
p = 0.0056
BRCA TAGLN2 PFI Survival
Gene=High expression (542)
Gene=Low expression (542)
p = 0.034
GBM TAGLN2 PFI Survival
Gene=High expression (83)
Gene=Low expression (82)
p = 0.036
KIRC TAGLN2 PFI Survival
Gene=High expression (264)
Gene=Low expression (263)
p = 0.0029
KIRP TAGLN2 PFI Survival
Gene=High expression (142)
Gene=Low expression (142)
p = 0.026
LGG TAGLN2 PFI Survival
Gene=High expression (259)
Gene=Low expression (259)
p < 0.0001
LIHC TAGLN2 PFI Survival
Gene=High expression (183)
Gene=Low expression (183)
p = 0.039
Survival probability
Time (days)
Number at risk
Strata

Supplementary Figure 3. The corresponding Kaplan-Meier curves regarding OS(A), DFI(B), DSS(C), and PFI(D).

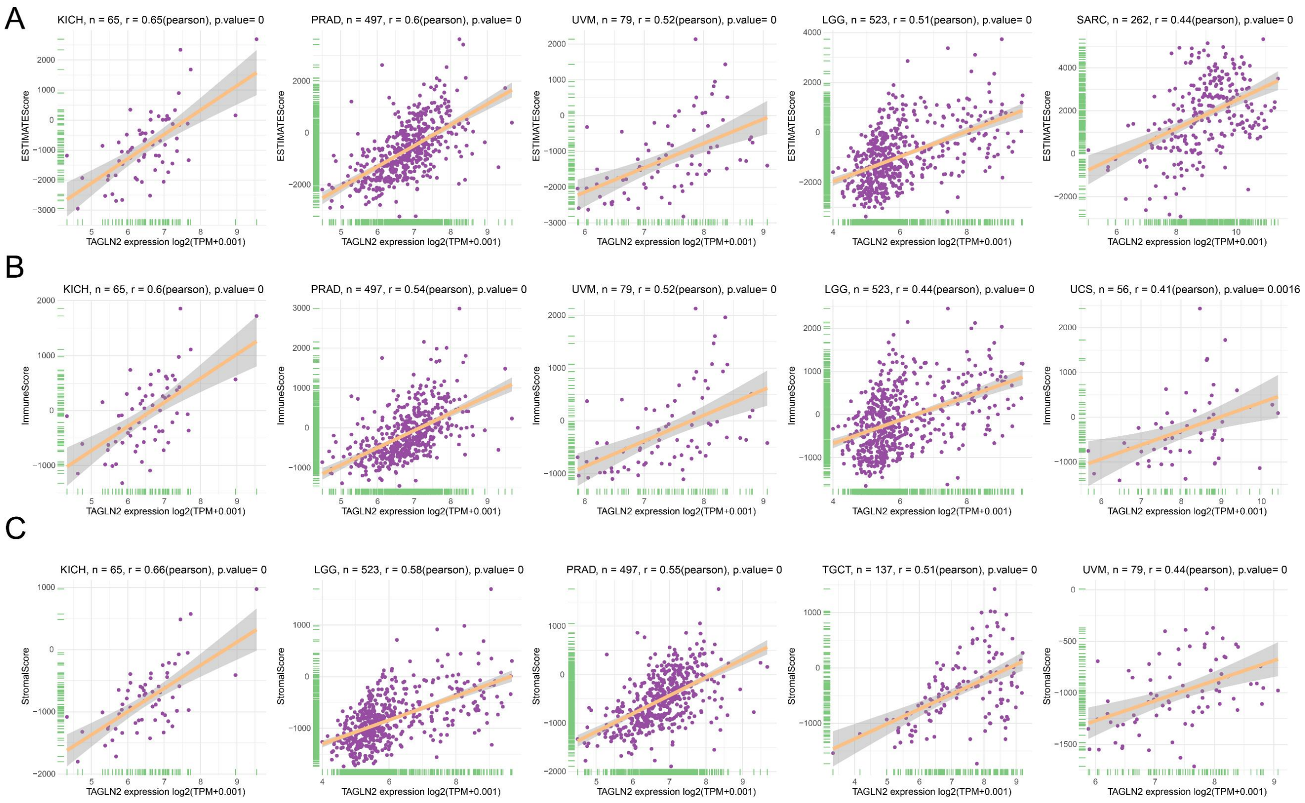

Supplementary Figure 4. The top 5 tumors with the most significant correlation between the degree of immune infiltration and TAGLN2 expression for ESTIMATEScore (A); for ImmuneScore(B); for StromalScore (C), solely.

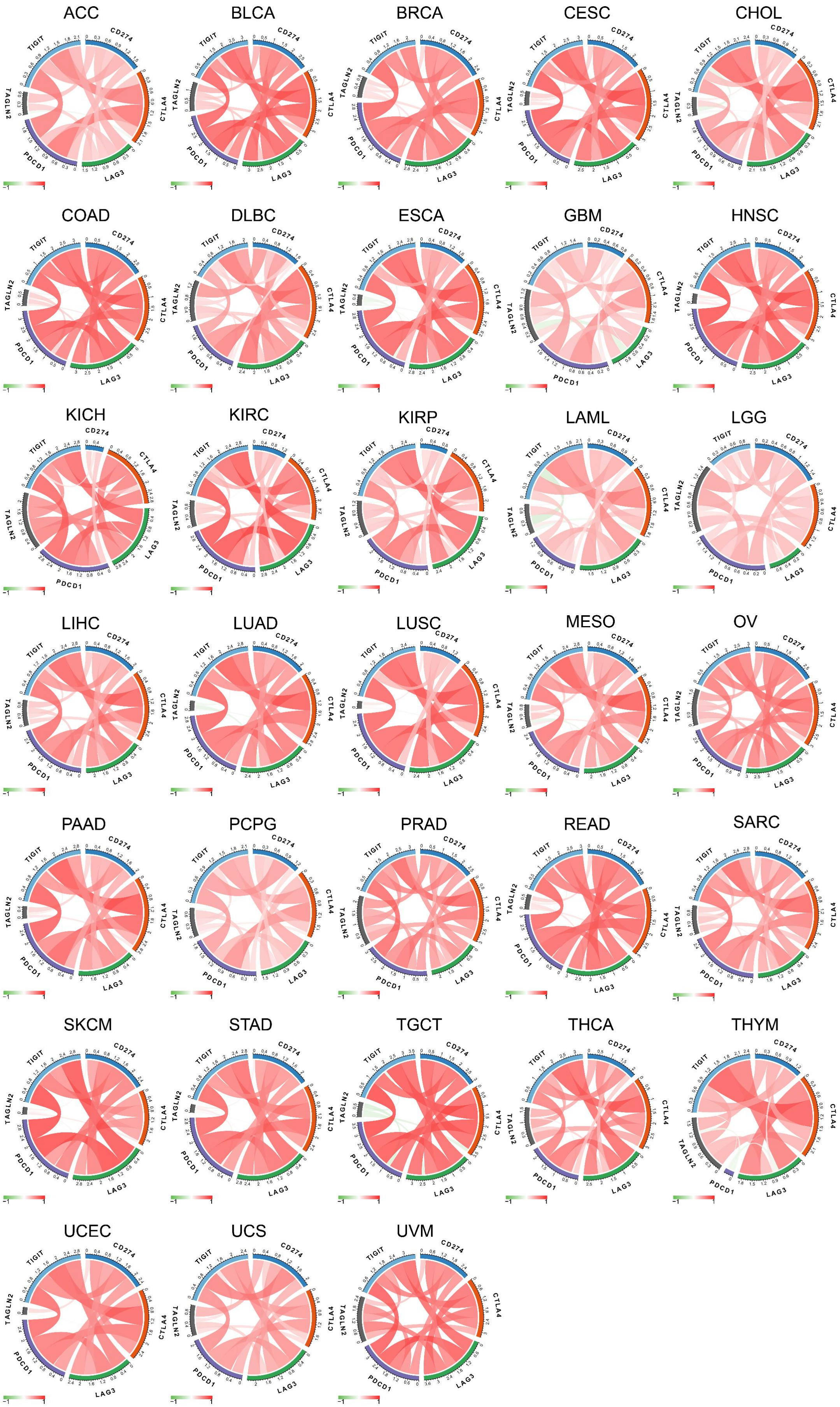
ACC
BLCA
BRCA
CESC
CHOL
COAD
DLBC
ESCA
GBM
HNSC
KICH
KIRC
KIRP
LAML
LGG
LIHC
LUAD
LUSC
MESO
OV
PAAD
PCPG
PRAD
READ
SARC
SKCM
STAD
TGCT
THCA
THYM
UCEC
UCS
UVM
CD274
CTLA4
LAG3
PDCD1
TAGLN2
TIGIT
-1
1

Supplementary Figure 5. Correlation analyses of the TAGLN2 expression with immune checkpoint genes in pan-cancer.

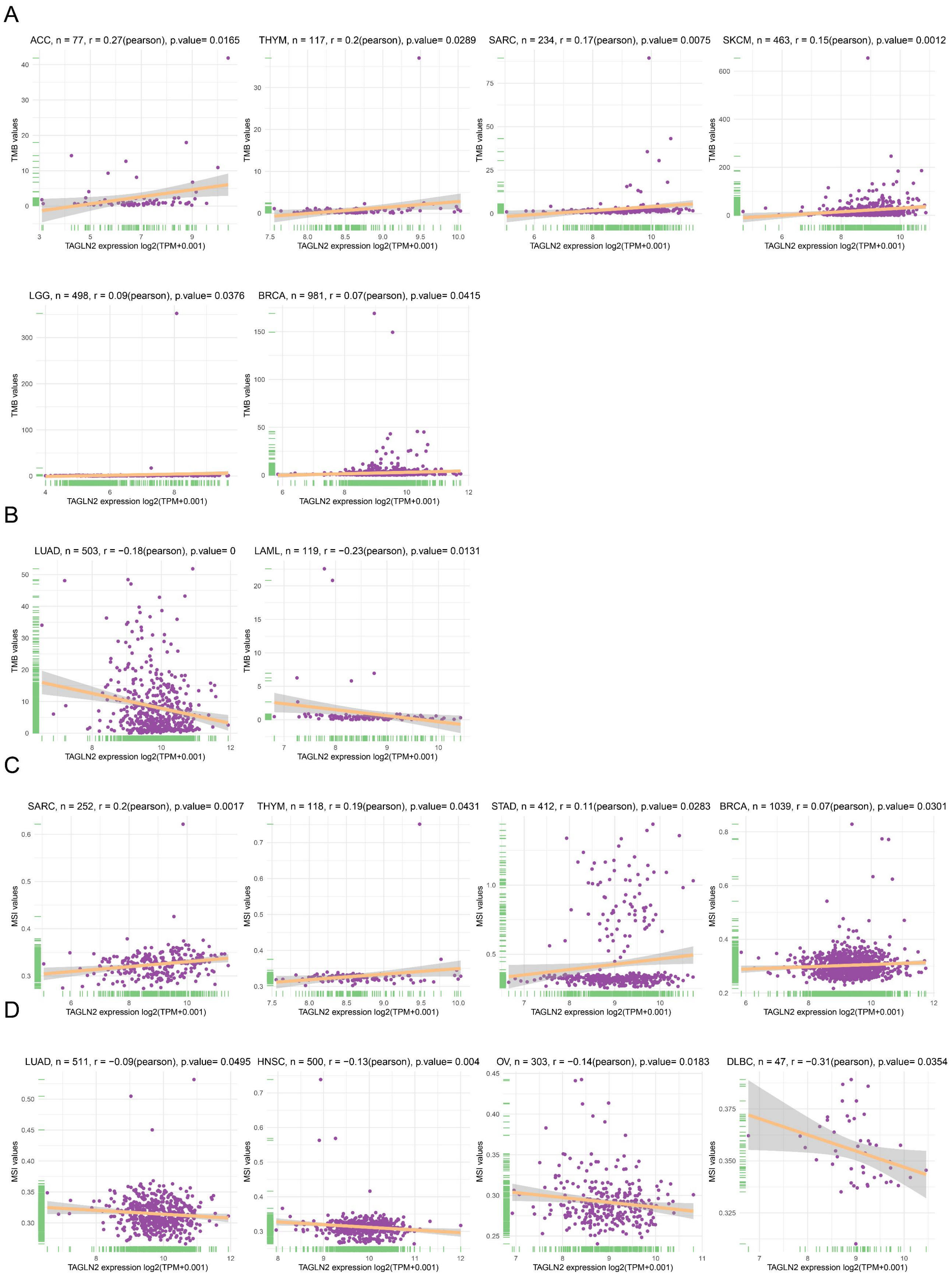
A
ACC, n = 77, r = 0.27(pearson), p.value= 0.0165
THYM, n = 117, r = 0.2(pearson), p.value= 0.0289
SARC, n = 234, r = 0.17(pearson), p.value= 0.0075
SKCM, n = 463, r = 0.15(pearson), p.value= 0.0012
LGG, n = 498, r = 0.09(pearson), p.value= 0.0376
BRCA, n = 981, r = 0.07(pearson), p.value= 0.0415
TMB values
TAGLN2 expression log2(TPM+0.001)
B
LUAD, n = 503, r = −0.18(pearson), p.value= 0
LAML, n = 119, r = −0.23(pearson), p.value= 0.0131
TMB values
TAGLN2 expression log2(TPM+0.001)
C
SARC, n = 252, r = 0.2(pearson), p.value= 0.0017
THYM, n = 118, r = 0.19(pearson), p.value= 0.0431
STAD, n = 412, r = 0.11(pearson), p.value= 0.0283
BRCA, n = 1039, r = 0.07(pearson), p.value= 0.0301
MSI values
TAGLN2 expression log2(TPM+0.001)
D
LUAD, n = 511, r = −0.09(pearson), p.value= 0.0495
HNSC, n = 500, r = −0.13(pearson), p.value= 0.004
OV, n = 303, r = −0.14(pearson), p.value= 0.0183
DLBC, n = 47, r = −0.31(pearson), p.value= 0.0354
MSI values
TAGLN2 expression log2(TPM+0.001)

Supplementary Figure 6. Scatter plots between TAGLN2 and TMB and MSI.

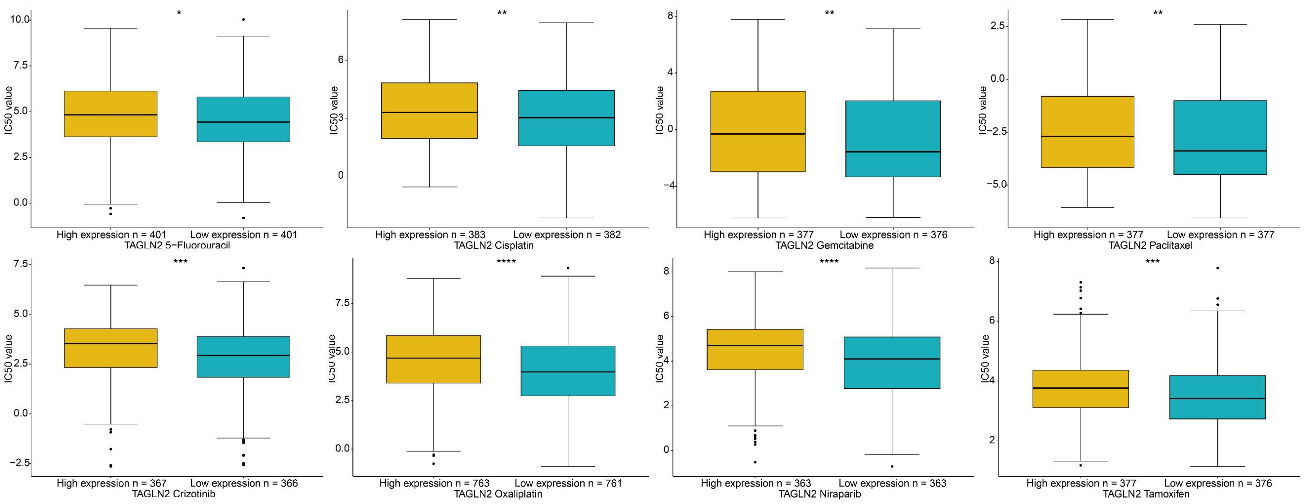

Supplementary Figure 7. An illustration of the relationship between TAGLN2 expression and common medication response. * p-value < 0.05, ** p-value < 0.01, *** p-value < 0.001, and **** p-value < 0.0001.

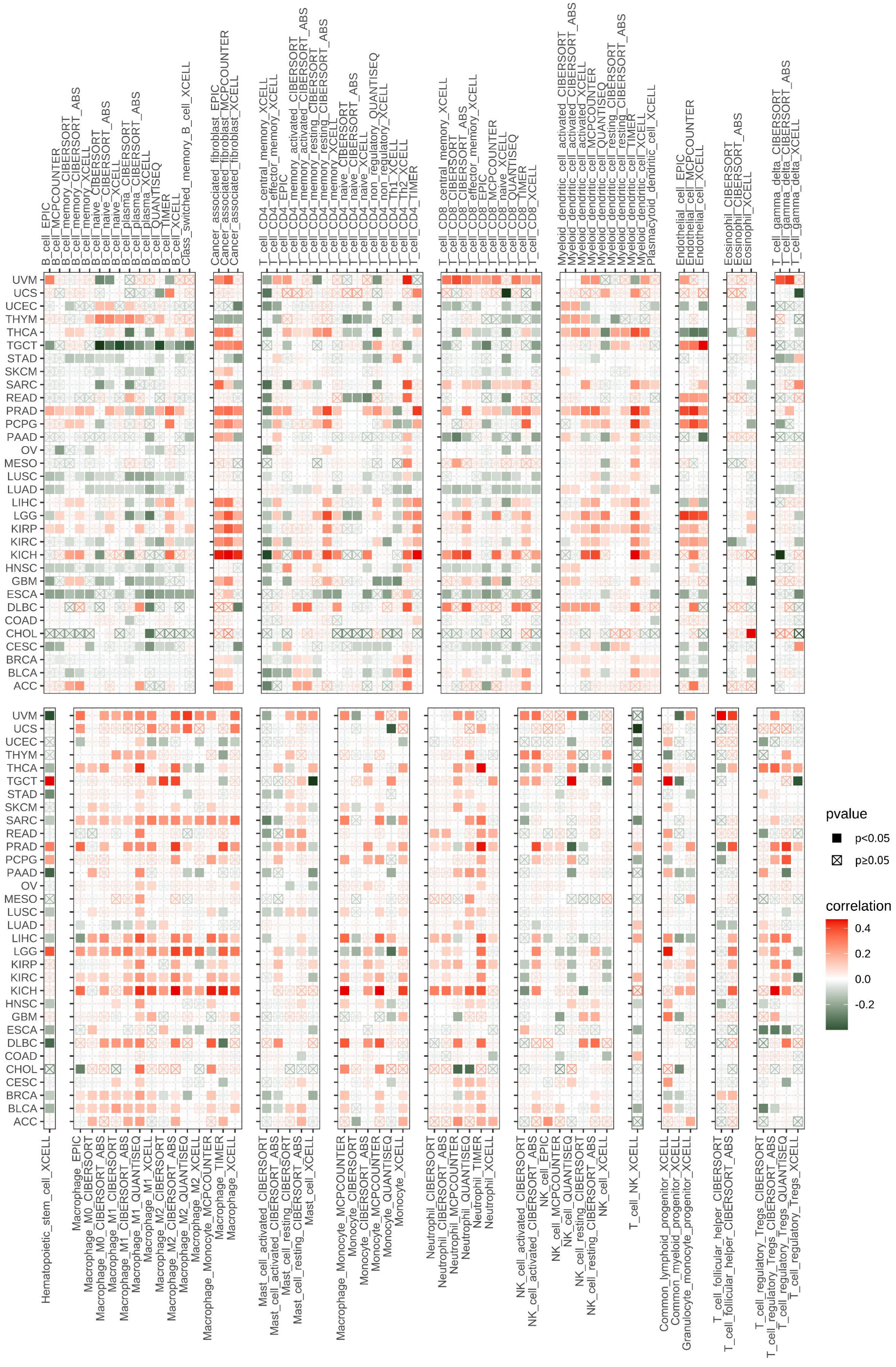
B_cell_EPIC
B_cell_MCPCOUNTER
B_cell_memory_CIBERSORT
B_cell_memory_CIBERSORT_ABS
B_cell_memory_XCELL
B_cell_naive_CIBERSORT
B_cell_naive_CIBERSORT_ABS
B_cell_naive_XCELL
B_cell_plasma_CIBERSORT
B_cell_plasma_CIBERSORT_ABS
B_cell_plasma_XCELL
B_cell_QUANTISEQ
B_cell_TIMER
B_cell_XCELL
Class_switched_memory_B_cell_XCELL
Cancer_associated_fibroblast_EPIC
Cancer_associated_fibroblast_MCPCOUNTER
Cancer_associated_fibroblast_XCELL
T_cell_CD4_central_memory_XCELL
T_cell_CD4_effector_memory_XCELL
T_cell_CD4_EPIC
T_cell_CD4_memory_activated_CIBERSORT
T_cell_CD4_memory_activated_CIBERSORT_ABS
T_cell_CD4_memory_resting_CIBERSORT
T_cell_CD4_memory_resting_CIBERSORT_ABS
T_cell_CD4_memory_XCELL
T_cell_CD4_naive_CIBERSORT
T_cell_CD4_naive_CIBERSORT_ABS
T_cell_CD4_naive_XCELL
T_cell_CD4_non_regulatory_QUANTISEQ
T_cell_CD4_non_regulatory_XCELL
T_cell_CD4_Th1_XCELL
T_cell_CD4_Th2_XCELL
T_cell_CD4_TIMER
T_cell_CD8_central_memory_XCELL
T_cell_CD8_CIBERSORT
T_cell_CD8_CIBERSORT_ABS
T_cell_CD8_effector_memory_XCELL
T_cell_CD8_EPIC
T_cell_CD8_MCPCOUNTER
T_cell_CD8_naive_XCELL
T_cell_CD8_QUANTISEQ
T_cell_CD8_TIMER
T_cell_CD8_XCELL
Myeloid_dendritic_cell_activated_CIBERSORT
Myeloid_dendritic_cell_activated_CIBERSORT_ABS
Myeloid_dendritic_cell_activated_XCELL
Myeloid_dendritic_cell_MCPCOUNTER
Myeloid_dendritic_cell_QUANTISEQ
Myeloid_dendritic_cell_resting_CIBERSORT
Myeloid_dendritic_cell_resting_CIBERSORT_ABS
Myeloid_dendritic_cell_TIMER
Myeloid_dendritic_cell_XCELL
Plasmacytoid_dendritic_cell_XCELL
Endothelial_cell_EPIC
Endothelial_cell_MCPCOUNTER
Endothelial_cell_XCELL
Eosinophil_CIBERSORT
Eosinophil_CIBERSORT_ABS
Eosinophil_XCELL
T_cell_gamma_delta_CIBERSORT
T_cell_gamma_delta_CIBERSORT_ABS
T_cell_gamma_delta_XCELL
UVM
UCS
UCEC
THYM
THCA
TGCT
STAD
SKCM
SARC
READ
PRAD
PCPG
PAAD
OV
MESO
LUSC
LUAD
LIHC
LGG
KIRP
KIRC
KICH
HNSC
GBM
ESCA
DLBC
COAD
CHOL
CESC
BRCA
BLCA
ACC
Hematopoietic_stem_cell_XCELL
Macrophage_EPIC
Macrophage_M0_CIBERSORT
Macrophage_M0_CIBERSORT_ABS
Macrophage_M1_CIBERSORT
Macrophage_M1_CIBERSORT_ABS
Macrophage_M1_QUANTISEQ
Macrophage_M1_XCELL
Macrophage_M2_CIBERSORT
Macrophage_M2_CIBERSORT_ABS
Macrophage_M2_QUANTISEQ
Macrophage_M2_XCELL
Macrophage_Monocyte_MCPCOUNTER
Macrophage_TIMER
Macrophage_XCELL
Mast_cell_activated_CIBERSORT
Mast_cell_activated_CIBERSORT_ABS
Mast_cell_resting_CIBERSORT
Mast_cell_resting_CIBERSORT_ABS
Mast_cell_XCELL
Macrophage_Monocyte_MCPCOUNTER
Monocyte_CIBERSORT
Monocyte_CIBERSORT_ABS
Monocyte_MCPCOUNTER
Monocyte_QUANTISEQ
Monocyte_XCELL
Neutrophil_CIBERSORT
Neutrophil_CIBERSORT_ABS
Neutrophil_MCPCOUNTER
Neutrophil_QUANTISEQ
Neutrophil_TIMER
Neutrophil_XCELL
NK_cell_activated_CIBERSORT
NK_cell_activated_CIBERSORT_ABS
NK_cell_EPIC
NK_cell_MCPCOUNTER
NK_cell_QUANTISEQ
NK_cell_resting_CIBERSORT
NK_cell_resting_CIBERSORT_ABS
NK_cell_XCELL
T_cell_NK_XCELL
Common_lymphoid_progenitor_XCELL
Common_myeloid_progenitor_XCELL
Granulocyte_monocyte_progenitor_XCELL
T_cell_follicular_helper_CIBERSORT
T_cell_follicular_helper_CIBERSORT_ABS
T_cell_regulatory_Tregs_CIBERSORT
T_cell_regulatory_Tregs_CIBERSORT_ABS
T_cell_regulatory_Tregs_QUANTISEQ
T_cell_regulatory_Tregs_XCELL
pvalue
p<0.05
p≥0.05
correlation
0.4
0.2
0.0
-0.2

Supplementary Figure 8. The relevance of TAGLN2 expression and immune cell infiltration in pan-cancer by using TIMER2.0 database.

Table S1. Summary of Spearman's correlation between TAGLN2 expression and drug resistance in cancer cell lines based upon the GDSC2 dataset.

**correlation**	**pvalue**	**type**	**label**
0.137431	9.25E-05	Camptothecin	positive
0.152999	2.68E-05	Vinblastine	positive
0.136433	0.0001537	Cisplatin	positive
0.224242	5.44E-10	Cytarabine	positive
0.088256	0.0008347	Docetaxel	positive
0.147182	5.39E-05	Navitoclax	positive
0.213021	3.39E-09	Vorinostat	positive
0.145396	6.15E-05	Nilotinib	positive
0.183712	3.52E-07	Olaparib	positive
0.148305	4.45E-05	Wee1 Inhibitor	positive
0.197397	3.40E-08	Nutlin-3a (-)	positive
0.186232	4.34E-07	Mirin	positive
0.118443	0.0011118	PD173074	positive
0.149247	5.35E-05	Alisertib	positive
0.100784	0.0052102	MK-2206	positive
0.149165	3.40E-05	Palbociclib	positive
0.082419	0.0240889	Dactolisib	positive
0.092062	0.0130808	Obatoclax Mesylate	positive
0.088717	0.0119553	5-Fluorouracil	positive
0.12063	0.0009034	Paclitaxel	positive
0.154655	2.61E-05	Crizotinib	positive
0.245945	7.51E-12	Sorafenib	positive
0.172575	9.38E-07	Irinotecan	positive
0.203272	1.12E-15	Oxaliplatin	positive
0.15266	1.48E-05	GSK1904529A	positive
0.14032	0.0001488	PRIMA-1MET	positive
0.191396	2.03E-07	Niraparib	positive
0.11217	0.0018757	MK-1775	positive
0.107171	0.0038644	Dinaciclib	positive
0.122979	0.0007199	Gemcitabine	positive
0.162673	7.24E-06	Tamoxifen	positive
0.124758	1.40E-06	Fulvestrant	positive
0.163295	3.54E-06	EPZ004777	positive
0.123364	0.0007157	YK-4-279	positive
0.272778	6.56E-08	Daporinad	positive
0.171239	3.48E-06	BMS-345541	positive
0.147975	5.02E-05	Talazoparib	positive
0.143158	8.07E-05	Dabrafenib	positive
0.15247	2.86E-05	Temozolomide	positive

0.119885	0.0012108	AZD5438	positive
0.122993	0.0009198	IAP_5620	positive
0.083007	0.0253146	AZD2014	positive
0.159464	1.60E-05	AZD1208	positive
0.11487	0.001935	Ruxolitinib	positive
0.191823	4.55E-08	Linsitinib	positive
0.172336	2.09E-06	Epirubicin	positive
0.156313	1.75E-05	Cyclophosphamide	positive
0.191385	1.36E-07	Pevonedistat	positive
0.087223	0.0008015	Uprosertib	positive
0.090227	0.0150906	LCL161	positive
0.158402	6.92E-06	EPZ5676	positive
0.114509	0.0020144	IWP-2	positive
0.171372	3.42E-06	Leflunomide	positive
0.147937	5.87E-05	Entinostat	positive
0.168194	1.72E-06	LGK974	positive
0.128343	0.0005273	VE-822	positive
0.090008	0.0152682	CZC24832	positive
0.102085	0.0063304	AZD5582	positive
0.108762	0.0035009	GSK2606414	positive
0.143212	0.0001079	PFI3	positive
0.185824	4.60E-07	PCI-34051	positive
0.165788	7.11E-06	Wnt-C59	positive
0.103449	0.0053012	GSK343	positive
0.193927	1.39E-07	ML323	positive
0.074045	0.04611	PRT062607	positive
0.186158	4.38E-07	AGI-6780	positive
0.118249	0.0014136	Picolinici-acid	positive
0.11348	0.0022122	CDK9_5576	positive
0.122581	0.0009418	CDK9_5038	positive
0.149407	5.57E-05	Eg5_9814	positive
0.108513	0.0034178	IRAK4_4710	positive
0.106853	0.0039467	JAK1_8709	positive
0.152164	4.03E-05	AZD5991	positive
0.105615	0.0044157	PAK_5339	positive
0.197734	7.96E-08	TAF1_5496	positive
0.082902	0.0257044	ULK1_4989	positive
0.095071	0.0104296	IGF1R_3801	positive
0.088826	0.0166664	JAK_8517	positive
0.147074	5.10E-05	AZD4547	positive
0.165315	7.55E-06	Zoledronate	positive
0.075661	0.041544	Acetalax	positive
0.181426	8.62E-07	Carmustine	positive
0.141853	0.0001256	Topotecan	positive
0.145048	8.77E-05	Teniposide	positive

0.157502	2.02E-05	Mitoxantrone	positive
0.117914	6.12E-06	Dactinomycin	positive
0.129214	0.0004826	Fludarabine	positive
0.123534	0.0008583	Nelarabine	positive
0.158993	1.82E-05	Vincristine	positive
0.119731	0.0012286	Podophyllotoxin bromide	positive
0.133832	0.0003049	Dihydrorotenone	positive
0.156643	2.24E-05	Gallibiscoquinazole	positive
0.164487	8.40E-06	Elephantin	positive
0.105677	0.0043653	Sinularin	positive
0.229682	4.02E-10	Sabutoclax	positive
0.144473	9.36E-05	LY2109761	positive
0.13952	0.0001624	OF-1	positive
0.188647	3.05E-07	KRAS (G12C) Inhibitor-12	positive
0.107543	0.0032102	Buparlisib	positive
0.116515	6.28E-06	Ulixertinib	positive
0.18423	3.57E-07	Venetoclax	positive
0.164427	6.18E-06	ABT737	positive
0.120307	0.0009778	Afuresertib	positive
0.147895	4.84E-05	AGI-5198	positive
0.101869	0.005262	AZD6738	positive
0.09985	0.0062386	Ipatasertib	positive
0.132733	0.0002698	GDC0810	positive
0.164623	5.93E-06	GSK2578215A	positive
0.134321	0.0002272	I-BRD9	positive
0.170128	2.83E-06	Telomerase Inhibitor IX	positive
0.182636	4.93E-07	MIRA-1	positive
0.134692	0.0002182	NVP-ADW742	positive
0.188901	1.91E-07	P22077	positive
0.135042	0.0002121	Savolitinib	positive
0.137423	0.0001616	UMI-77	positive
0.094377	0.009756	Sepantronium bromide	positive
0.152233	2.87E-05	MIM1	positive
0.127397	0.0004824	Foretinib	positive
0.168599	3.65E-06	BIBR-1532	positive
0.120319	0.0009844	Pyridostatin	positive
0.076503	0.0369544	AMG-319	positive
0.108095	0.0031155	MK-8776	positive
0.138917	0.000141	Vinorelbine	positive
0.113854	0.0018815	LJI308	positive
0.102372	0.0052207	AZ6102	positive
0.078684	0.0321095	GSK591	positive
0.098878	0.0070674	VE821	positive
**correlation**	**pvalue**	**type**	**label**
-0.101338	0.0049105	Staurosporine	negtive

-0.201365	2.29E-08	Dasatinib	negtive
-0.094647	0.0073875	Sapitinib	negtive
**correlation**	**pvalue**	**type**	**label**
0.031573	0.3882146	Gefitinib	non-significant
-0.132653	0.3635483	Axitinib	non-significant
0.046751	0.197951	AZD7762	non-significant
0.184417	0.0767918	SB216763	non-significant
-0.144485	0.327189	KU-55933	non-significant
0.028036	0.4281334	Afatinib	non-significant
0.053247	0.1338516	PLX-4720	non-significant
-0.038853	0.7954134	NU7441	non-significant
0.013506	0.9290141	Doramapimod	non-significant
-0.281568	0.0551941	ZM447439	non-significant
0.006188	0.9667066	RO-3306	non-significant
0.038826	0.2841218	Pictilisib	non-significant
0.086763	0.408264	AZD8055	non-significant
0.037441	0.2898823	PD0325901	non-significant
0.057549	0.1172829	Rapamycin	non-significant
-0.075657	0.629672	BI-2536	non-significant
-0.017571	0.6311546	BMS-536924	non-significant
-0.198437	0.1763559	Tozasertib	non-significant
0.116261	0.4313262	PF-4708671	non-significant
-0.010172	0.7810682	Erlotinib	non-significant
-0.024843	0.4960777	Bortezomib	non-significant
0.005319	0.9713773	GSK269962A	non-significant
-0.162187	0.2871316	SB505124	non-significant
0.058901	0.1135601	AZ960	non-significant
0.012054	0.7457577	XAV939	non-significant
-0.065599	0.0694099	Trametinib	non-significant
-0.013026	0.7260523	AZD1332	non-significant
0.015393	0.6730301	Lapatinib	non-significant
0.056956	0.1072359	Luminespib	non-significant
0.064432	0.0683655	Alpelisib	non-significant
0.027209	0.4418907	Taselisib	non-significant
9.09E-05	0.997952	SCH772984	non-significant
-0.118076	0.0562912	OSI-027	non-significant
0.05742	0.1221625	WZ4003	non-significant
-0.016919	0.6492489	I-BET-762	non-significant
0.034926	0.3476916	RVX-208	non-significant
-0.030087	0.4182479	OTX015	non-significant
-0.044177	0.2345019	Entospletinib	non-significant
0.121061	0.4228819	Ribociclib	non-significant
0.027182	0.4649155	AZD5153	non-significant
-0.026554	0.4749902	ERK_2440	non-significant
0.010731	0.7728548	ERK_6604	non-significant

0.048352	0.1931427	VSP34_8731	non-significant
-0.030827	0.4068958	Selumetinib	non-significant
-0.018258	0.6242955	Ibrutinib	non-significant
0.004237	0.9092679	MN-64	non-significant
-0.016518	0.6474281	MG-132	non-significant
0.043447	0.2349834	BDP-00009066	non-significant
-0.00884	0.8081476	AZD3759	non-significant
0.062201	0.0889218	AZD5363	non-significant
-0.024534	0.5025893	AZD8186	non-significant
0.053999	0.1398236	Osimertinib	non-significant
0.052995	0.1473524	Cediranib	non-significant
0.056126	0.1284556	GNE-317	non-significant
0.016584	0.6506734	WIKI4	non-significant
0.058019	0.1126142	WEHI-539	non-significant
0.004481	0.9025659	BPD-00008900	non-significant
0.051328	0.1622154	VX-11e	non-significant
-0.015957	0.9152177	AZD6482	non-significant
0.052334	0.1544064	AT13148	non-significant
-0.085271	0.5820817	BMS-754807	non-significant
0.095976	0.5210393	JQ1	non-significant

Table S1. Summary of Spearman's correlation between TAGLN2 expression and drug resistance in cancer cell lines based upon the GDSC2 dataset.

**Table S2. The sample size information.**				
**Tumor type**	**Full names**	**Sample size of tumor tissues from TCGA**	**Sample size of normal tissues from TCGA**	**Sample size of normal tissues from GTEx**
ACC	Adrenocortical carcinoma	77	0	128
BLCA	Bladder Urothelial Carcinoma	407	19	9
BRCA	Breast invasive carcinoma	1098	113	179
CESC	Cervical squamous cell carcinoma and endocervical adenocarcinoma	306	3	10
CHOL	Cholangiocarcinoma	36	9	0
COAD	Colon adenocarcinoma	288	41	308
DLBC	Lymphoid Neoplasm Diffuse Large B-cell Lymphoma	47	0	444
ESCA	Esophageal carcinoma	182	13	653
GBM	Glioblastoma multiforme	165	0	1152
HNSC	Head and Neck squamous cell carcinoma	520	44	0
KICH	Kidney Chromophobe	66	25	28
KIRC	Kidney renal clear cell carcinoma	531	72	28
KIRP	Kidney renal papillary cell carcinoma	289	32	28

LAML	Acute Myeloid Leukemia	173	0	70
LGG	Brain Lower Grade Glioma	522	0	1152
LIHC	Liver hepatocellular carcinoma	371	50	110
LUAD	Lung adenocarcinoma	515	59	288
LUSC	Lung squamous cell carcinoma	498	50	288
MESO	Mesothelioma	87	0	0
OV	Ovarian serous cystadenocarcinoma	427	0	88
PAAD	Pancreatic adenocarcinoma	179	4	167
PCPG	Pheochromocytoma and Paraganglioma	182	3	0
PRAD	Prostate adenocarcinoma	496	52	100
READ	Rectum adenocarcinoma	92	10	308
SARC	Sarcoma	262	2	0
SKCM	Skin Cutaneous Melanoma	469	1	812
STAD	Stomach adenocarcinoma	414	36	174
TGCT	Testicular Germ Cell Tumors	137	0	165
THCA	Thyroid carcinoma	512	59	279
THYM	Thymoma	119	2	444
UCEC	Uterine Corpus Endometrial Carcinoma	181	13	89
UCS	Uterine Carcinosarcoma	57	0	89
UVM	Uveal Melanoma	79	0	0

Table S2. The sample size information.
